# Designer ^64^Cu Porphyrin–Trastuzumab Conjugates for Imaging of HER2-Positive Breast Cancer

**DOI:** 10.1021/cbmi.6c00024

**Published:** 2026-06-09

**Authors:** Peter C. Lawing, Hailey A. Houson, Zhiyuan Wu, Quang-Thien Ngo, Jonathan S. Lindsey, Suzanne E. Lapi

**Affiliations:** † Department of Chemistry, 6798 North Carolina State University, Raleigh, North Carolina 27695-8204, United States; ‡ Department of Radiology, Heersink School of Medicine, 9968 University of Alabama at Birmingham, Birmingham, Alabama 35294, United States; § Oncurie, Inc., Raleigh, North Carolina 27608, United States

**Keywords:** breast cancer, copper-64, HER2 imaging, porphyrin, positron emission tomography, synthesis, trastuzumab

## Abstract

The human epidermal growth factor receptor 2 (HER2) is overexpressed in 15–30% of breast cancer cases, plays a significant role in pathogenesis, and has been used as a diagnostic indicator and therapeutic target. Herein, a bioconjugatable porphyrin chelator is described, bearing a water-solubilizing swallowtail motif that projects above and below the plane of the macrocycle. This porphyrin, in various quantities, was reacted in stochastic fashion with the lysine side chains of the anti-HER2 antibody trastuzumab to afford a series of porphyrin–trastuzumab (**PorTraz**) products with conjugation ratios of 2.3, 3.5, 4.1, and 5.2. Radiolabeling of the conjugates with ^64^Cu in HEPES buffer (pH 7.2) at 37 °C afforded a molar activity of up to 20 μCi/μg (110 MBq/nmol). Binding was specific to the HER2 receptor, and uptake was observed for each conjugate. In mice bearing HER2+ BT-474 xenografts, PET imaging studies showed significantly higher tumor uptake, with a tumor standardized uptake value (SUV) of 0.95 ± 0.08 versus 0.38 ± 0.12 in the HER2– MDA-MB-468 xenograft control group at 24 h post-injection. PorTraz conjugates showed tumor SUV results of 1.25 ± 0.31 for n = 2.3 porphyrin chelators per antibody and 0.59 ± 0.20 for n = 5.2 porphyrin chelators per antibody. In summary, a series of porphyrin–trastuzumab conjugates were synthesized and labeled with ^64^Cu, displayed high binding to HER2+ cells, and illustrated uptake in mouse tumor xenografts observable by PET/CT imaging.

## Graphical abstract

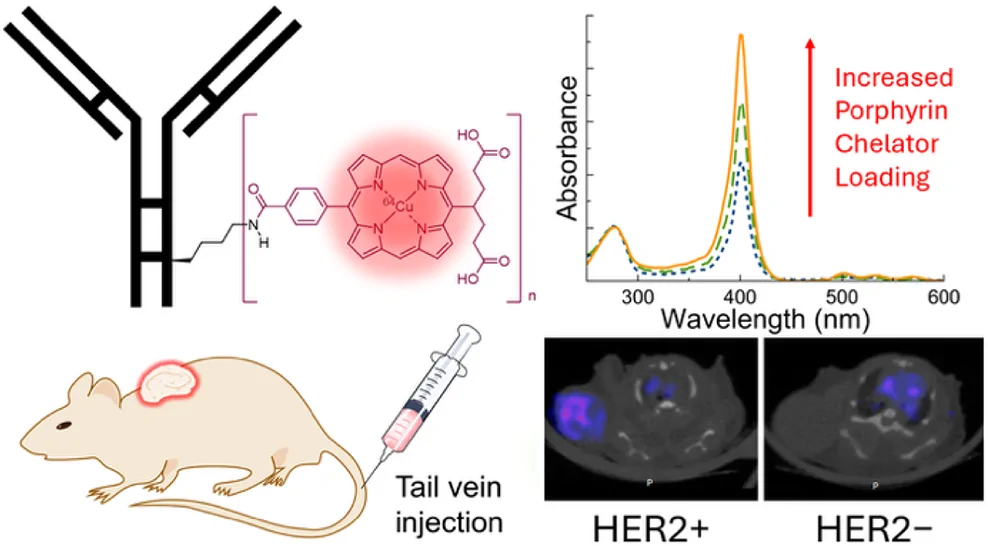

## Introduction

Breast cancer remains a significant healthcare challenge, estimated to account for more than 300,000 new cases and 42,000 deaths in the United States during 2025 alone.^1^ Biomarkers such as the estrogen receptor, the progesterone receptor, and the human epidermal growth factor receptor 2 (HER2) are used to classify breast cancer due to their influence over key factors such as cancer aggression and general prognosis.^2–4^ HER2 is overexpressed in 15–30% of breast cancer cases and typically coincides with more aggressive disease and a worsened prognosis,^5^ yet there has been great success over the last generation in the development of targeted therapies for HER2.^6^

One treatment for HER2+ breast cancer is trastuzumab, approved by the US FDA in 1998 as Herceptin, which inhibits the HER3/PI3K/AKT pathway; the inhibition directly correlates with *in vitro* antiproliferative effects on HER2+ cells.^7^ Trastuzumab binds to the FcγIII subunit of the HER2 surface protein, thereby disrupting the activation and inhibition of cell signaling pathways that control tumor growth.^8^ Trastuzumab, in conjunction with chemotherapy, improved median survival compared to chemotherapy alone.^9^ In 2013, the FDA approved the antibody–drug conjugate Ado-Trastuzumab emtansine (Kadcyla), which carries an average of 3.6 emtansine molecules per antibody.^10^ Emtansine, a derivative of maytansine, inhibits microtubule assembly in cancer cells.^11^ The selective delivery of this chemotherapeutic agent improves progression-free survival and overall survival with lower toxicity compared to a standard HER2+ chemotherapy regimen of lapatinib plus capecitabine.^12^ Furthermore, Kadcyla retains the clinical benefits of the trastuzumab antibody alone, and the binding affinity of the antibody remains unchanged with modification.^13^

Several groups have exploited the specific targeting properties of Trastuzumab for the development of HER2 imaging agents. In particular, clinical trials with^89^Zr-Trastuzumab have illustrated the capability to differentiate HER2+ from HER2– disease, provided deeper insights concerning HER2 heterogeneity, and, in some studies, have predicted likely therapeutic response.^14–18^

Recently, studies have illustrated the impact of chelator modifications on the biodistribution of the final antibody or other protein constructs for both target and nontarget tissues,^19–21^ illustrating the need for further development of suitable chelators specific to the intended application. Several groups have explored ^64^Cu-radiolabeled versions of trastuzumab, which show similarly promising results.^22–24^ The majority of these studies employed chelators that are relatively nonspecific for ^64^Cu. The development of a ^64^Cu-specific chelator–trastuzumab construct may potentially pave the way for therapeutic studies with the isotope ^67^Cu.

The metal-chelation features of porphyrins have made this class of molecules attractive for use in radiotherapy, and indeed, a flurry of research on porphyrins covalently tethered to antibodies occurred in the period 1987–1997.^25^ The porphyrins examined in that era generally were native-derived (e.g., from heme) or synthetic *meso*-tetraarylporphyrins, which often contained multiple sites of conjugation or unwanted bulky substituents. Subsequent advances in porphyrin synthetic methodology now enable the creation of designer porphyrins tailored for specific applications. A design of interest for biomedical applications utilizes sparsely substituted porphyrins,^26^ wherein only two substituents are employed: one for aqueous solubilization and one for bioconjugation. In this manner, the desired metal-chelation properties of porphyrins are exploited in a minimalistic design. A potent motif for aqueous solubilization is a derivatized swallowtail moiety, which is a symmetrically substituted alkyl unit bearing a polar group at each terminus. Upon attachment of a swallowtail unit at the meso-position of a porphyrin, steric factors cause the two alkyl groups to project largely perpendicular to the macrocycle plane.^27^ The projection of polar groups above and below the hydrophobic disk-shaped macrocycle imparts water solubility by suppressing self-aggregation (Figure 1). We recently studied the radiocupration of porphyrins, including those bearing swallowtail substituents, and found the porphyrins selectively underwent facile chelation with copper and were not demetalated under physiological conditions.^28^ Moreover, the porphyrins preferentially chelated copper even upon presentation with a metalation cocktail comprised of Fe­(II), Fe­(III), Co­(II), Ni­(II), Cu­(II), and Zn­(II). The preferential metalation with Cu­(II) is attractive given that sources of radiometals typically contain a variety of adventitious metals.^28^

**Figure 1:**
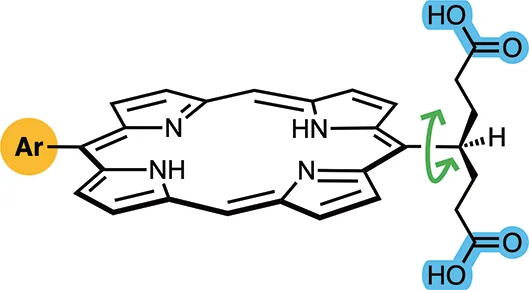
A swallowtail motif for aqueous solubilization of a porphyrin.

The present work describes the synthesis of a sparsely substituted, water-soluble, bioconjugatable porphyrin and the preparation of conjugates thereof with the trastuzumab antibody. Because of the stochastic nature of the bioconjugation reaction, each **PorTraz** conjugate is a rich mixture containing different numbers of porphyrin chelators, in addition to the underlying diversity due to antibody *N*-linked glycosylation.^29^ Each mixture has been characterized by ultraviolet–visible (UV–vis) absorption spectroscopy, matrix-assisted laser desorption ionization mass spectrometry (MALDI-MS), and capillary electrophoresis mass spectrometry (CE-MS). The conjugates were metalated with copper-64 under mild conditions for *in vitro* and *in vivo* studies in HER2+ breast cancer cell lines and animal models. Taken together, the synthesis, facile characterization, and biological studies show the utility of porphyrins as chelators of radiocopper for use in theranostics.

## Results and Discussion

### Synthesis of a Water-Soluble Bioconjugatable Porphyrin

The synthesis of the water-soluble bioconjugatable porphyrin (**Por-NHS**) began with the statistical condensation of three components: dipyrromethane (**1**),^30^ *N*-hydroxysuccinimidyl (NHS)-ester benzaldehyde **2**,^31^ and swallowtail-dimethyl acetal **3** ^27^ (Scheme 1, upper panel). In general, the statistical reaction of dipyrromethane and aldehydes A and B gives a mixture composed of a *trans*-A_2_-porphyrin, *trans-*AB-porphyrin, and *trans*-B_2_-porphyrin in a 1:2:1 ratio, respectively. The use of an acetal in lieu of an aldehyde for porphyrin formation has been reported over the years.^32–34^ Thus, condensation of dipyrromethane (**1**), benzaldehyde **2**, and swallowtail dimethyl acetal **3** in dichloromethane containing trifluoroacetic acid (TFA) at room temperature, followed by oxidation with DDQ, afforded, upon chromatographic purification, the target *trans*-AB-porphyrin **4** in 16% overall yield. By contrast, a similar synthesis using 4-carboxybenzaldehyde (instead of the NHS-ester analogue **2**) and the aldehyde analogue of **3** gave the corresponding *trans*-AB-porphyrin in only 5% yield.^27^ Subsequent removal of the 2-(trimethylsilyl)­ethyl group of **4** upon treatment with TFA^35^ afforded the desired **Por-NHS** in a 10-mg quantity. The porphyrin contains a single swallowtail group, wherein each alkyl unit is terminated with a carboxylic acid. The new porphyrins **4** and **Por-NHS** were characterized by ^1^H and ^13^C­{^1^H} NMR spectroscopy, UV–vis absorption spectroscopy, MALDI-MS, and electrospray ionization mass spectrometry (ESI-MS).

**Scheme 1:**
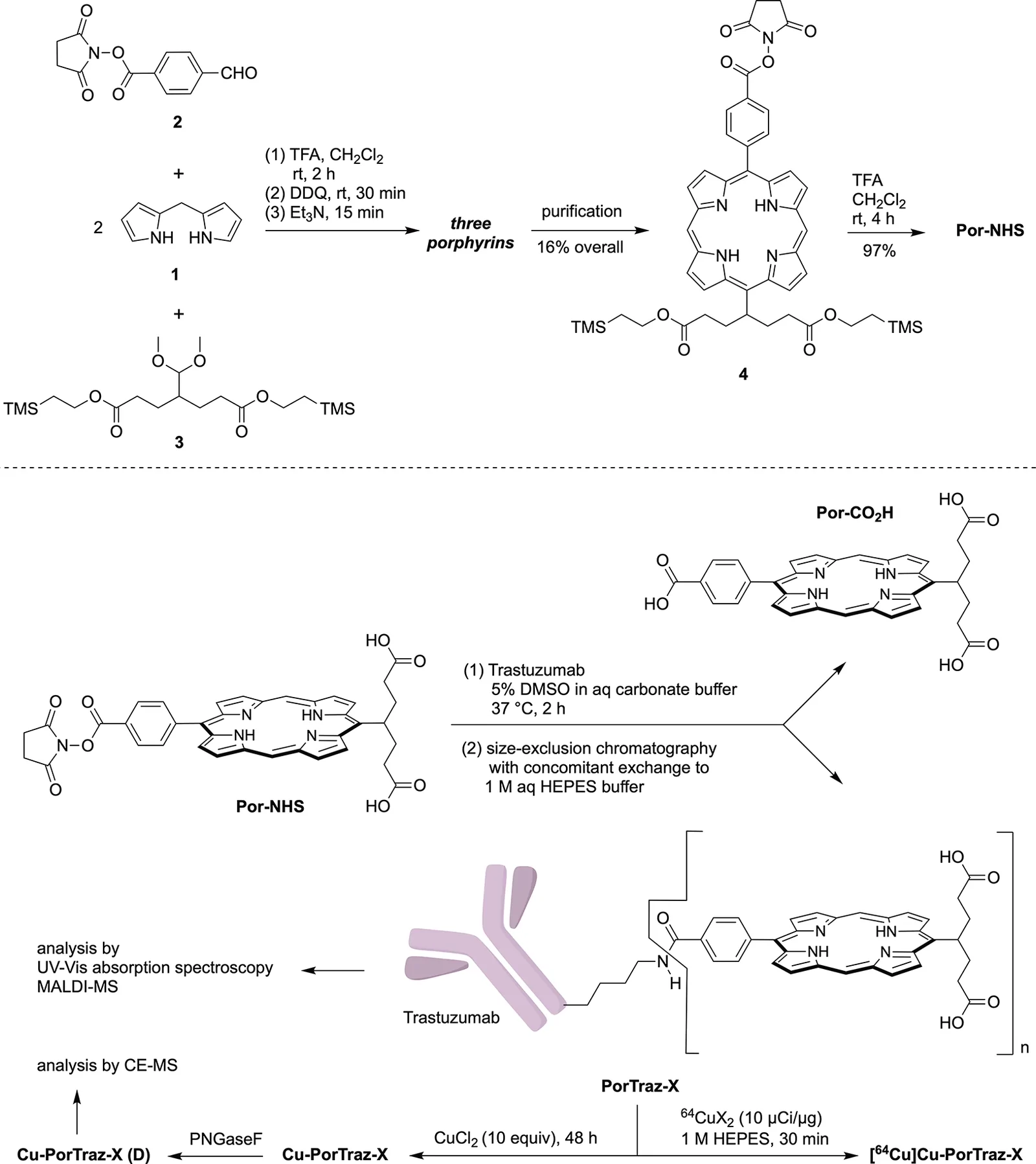
Synthesis of the Swallowtail Porphyrin NHS-Ester **Por-NHS** (Upper Panel) and Porphyrin–Trastuzumab Conjugates (Lower Panel)

### Porphyrin–Antibody Conjugation and Purification

Several porphyrin–trastuzumab conjugates were prepared by the reaction of **Por-NHS** with the lysine side chain or *N*-terminus^36–38^ of trastuzumab (Scheme 1, lower panel). The reactions were carried out in an aqueous NaHCO_3_/Na_2_CO_3_ buffer solution (pH 9, hereafter termed “aq carbonate”) containing 5% dimethyl sulfoxide (DMSO) for 2 h at 37 °C, conditions that were recently developed for high-yield conjugation.^39^ The ratio of **Por-NHS** to trastuzumab (130 μM) in the reaction mixture was varied from 3.5:1 to 14:1 to afford the porphyrin–trastuzumab samples (**PorTraz-I**, **II**, **III**, **IV**, and **V**) with various porphyrin-to-trastuzumab conjugation ratios. Purification was performed using a size-exclusion chromatography column under centrifugal action. Unreacted **Por-NHS** and porphyrin byproducts, including the major hydrolysis product porphyrin–triacid^27^ **Por-CO**_**2**_ **H**, were removed. The purification process also resulted in a desirable exchange of buffers, from aq carbonate containing 5% DMSO to 1 M 2-(4-(2-hydroxyethyl)­piperazin-1-yl)­ethanesulfonic acid (HEPES) buffer at pH 7.2 (hereafter termed “aq HEPES”). The characterization of the porphyrin–trastuzumab samples is described in the next section.

### Porphyrin–Antibody Analysis

#### Absorption Spectroscopy

UV–vis absorption spectroscopy was employed for the characterization of porphyrin–trastuzumab conjugates. The absorption spectra of trastuzumab and the porphyrin **Por-CO**_**2**_ **H** in aq HEPES are provided in Figure 2A. Trastuzumab absorbs strongly at 280 nm (ε = 225,000 M^–1^cm^–1^)^40^ but essentially not at all at wavelengths >300 nm. The porphyrin absorbs strongly in the Soret band^41^ (401–408 nm; ε = 250,000 M^–1^cm^–1^ (401–408 nm)) but only weakly at 280 nm (ε = 6,000–8,000 M^–1^cm^–1^; see Table S1). The absorption spectrum of **Por-CO**_**2**_ **H** was found to be essentially unchanged in different solvent systems, including aq HEPES; acetonitrile with various compositions of water (25:75, 50:50, and 75:25); and DMSO (Figure S1, Table S1). The distinct absorption spectra and known (or measured) values of the molar absorption coefficients of the two constituents enable the quantitative assessment of the conjugation ratio (i.e., the average number of porphyrin chelators attached to the antibody).

**Figure 2:**
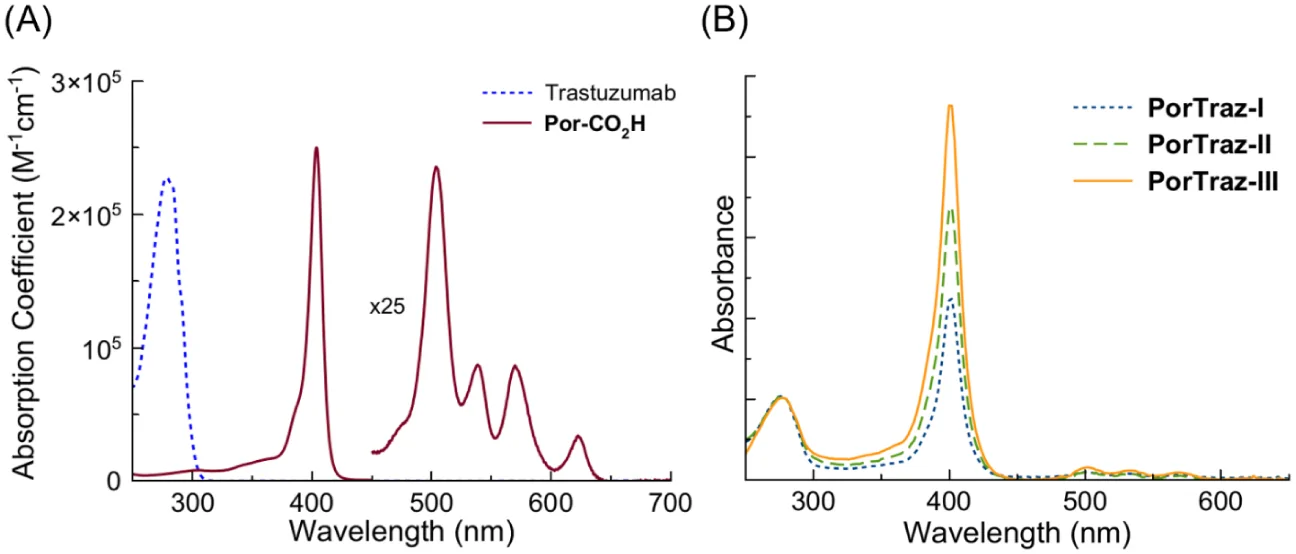
(A) Absorption spectra of trastuzumab and **Por-CO_2_H** in aq HEPES at room temperature. (B) UV–vis absorption spectra of **PorTraz** conjugates in aqueous acetonitrile upon HPLC elution (see text).

Three porphyrin–trastuzumab conjugates (**PorTraz-I**, **PorTraz-II**, and **PorTraz-III**) were analyzed using UV–vis absorption spectroscopy on three different instruments at different sample concentrations: (1) Nanodrop One Spectrophotometer (7–12 mg/mL), (2) Agilent 8453 Spectrophotometer (250 μg/mL in 1 cm quartz cuvettes), and (3) high-performance liquid chromatography–diode array detector (HPLC-DAD) instrument (5 μL of a 2 mg/mL sample was injected). The HPLC chromatography conditions employed 80 °C. The spectra for the three conjugates on the three instruments are shown in Figure S2. The most dilute solutions (HPLC-DAD analysis) showed no signs of aggregation or light scattering^42^ and were used for the determination of the conjugation ratios (Figures 2B and S3, Table S2 and S3). The average conjugation ratio of **PorTraz-I**, **PorTraz-II**, and **PorTraz-III** was found to be 2.3, 3.5, and 5.2 porphyrin chelators per antibody, respectively.

#### Mass Spectrometry

The porphyrin–trastuzumab conjugates **PorTraz-I**, **PorTraz-II**, and **PorTraz-III** were analyzed by MALDI-MS, a low-resolution but readily accessible method. While peaks due to incremental numbers of porphyrins per antibody (free base porphyrin, net 570 Da; trastuzumab, ∼148 500 Da) could not be detected, a clear shift in the mass-to-charge ratio (*m*/*z*) for the conjugates synthesized from a higher porphyrin–trastuzumab ratio was observed (Figure 3A and Table S4), consistent with the conjugation ratios observed by absorption spectroscopy.

**Figure 3:**
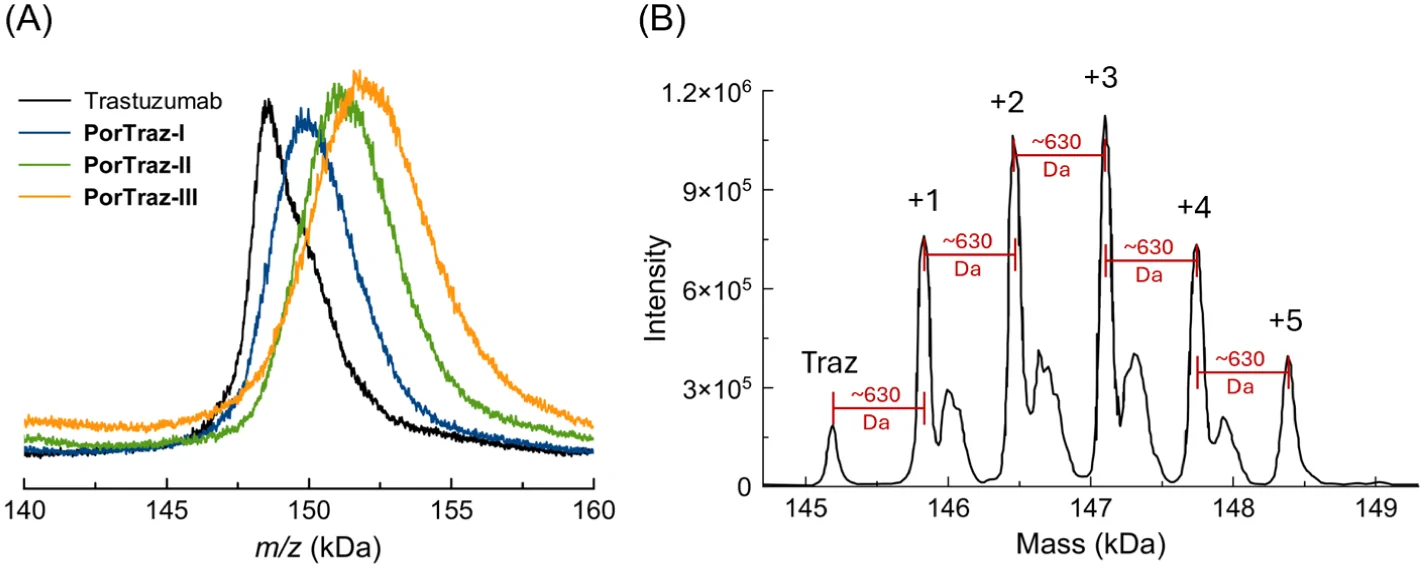
(A) MALDI-MS spectra of trastuzumab and three **PorTraz** conjugates. (B) CE-MS results for **Cu-PorTraz-IV**.

A porphyrin–trastuzumab conjugate, **PorTraz-IV** (conjugation ratio of 3.5 porphyrin chelators per antibody), was treated overnight with 10 equiv of copper­(II) chloride (per porphyrin) at 37 °C. Exhaustive cupration to form **Cu-PorTraz-IV** was confirmed by the absence of fluorescence^26^ at 600–750 nm from the free-base porphyrin.

While trastuzumab is monoclonal, diverse *N*-linked glycosylation on the F_c_ region^43^ affords a distribution of nonidentical masses. One approach to increase spectral clarity is to remove the glycosyl groups by enzymatic treatment. Treatment of trastuzumab with the enzyme PNGase F has resulted in deglycosylation, as reported by Zhu et al.^29^ The deglycosylation approach was repeated here with analysis by CE-MS (Figure S4), which showed a major peak from the trastuzumab antibody (M) and a minor peak (M + 162) representing trastuzumab bearing a hexose that was not removed in the deglycosylation process. In the same manner, deglycosylation of **Cu-PorTraz-IV** afforded **Cu-PorTraz-IV (D)**. CE-MS analysis showed six major peaks in the mass spectrum separated by ∼630 Da, which corresponds to the mass of the copper porphyrin (Figure 3B). A minor peak (M + 162) observed in both the deglycosylated trastuzumab and **Cu-PorTraz-IV (D)** samples corresponds to a hexose moiety that remains intact through the deglycosylation process. The six major peaks represent those antibodies in the mixture bearing 0, 1, 2, 3, 4, and 5 porphyrins, which is expected given the 88 lysine amines of the antibody and the Poissonian nature of the conjugation.^44^

### Porphyrin–Antibody Metalation Biological Characterization

Each **PorTraz** conjugate was radiolabeled with copper-64 (*t*_1/2_ = 12.7 h) at >90% radiochemical yield under standard conditions^26^ at the University of Alabama at Birmingham prior to additional studies. The radiochemical purity decreased as the specific activity increased. At a specific activity of 100 μCi/μg, the radiochemical purity was observed to decrease in the order **PorTraz-III** > **PorTraz-II** > **PorTraz-I**. This result is expected because the presence of a larger number of porphyrin molecules enables more copper to be loaded onto the porphyrin–antibody conjugate (Figure 4A).

**Figure 4:**
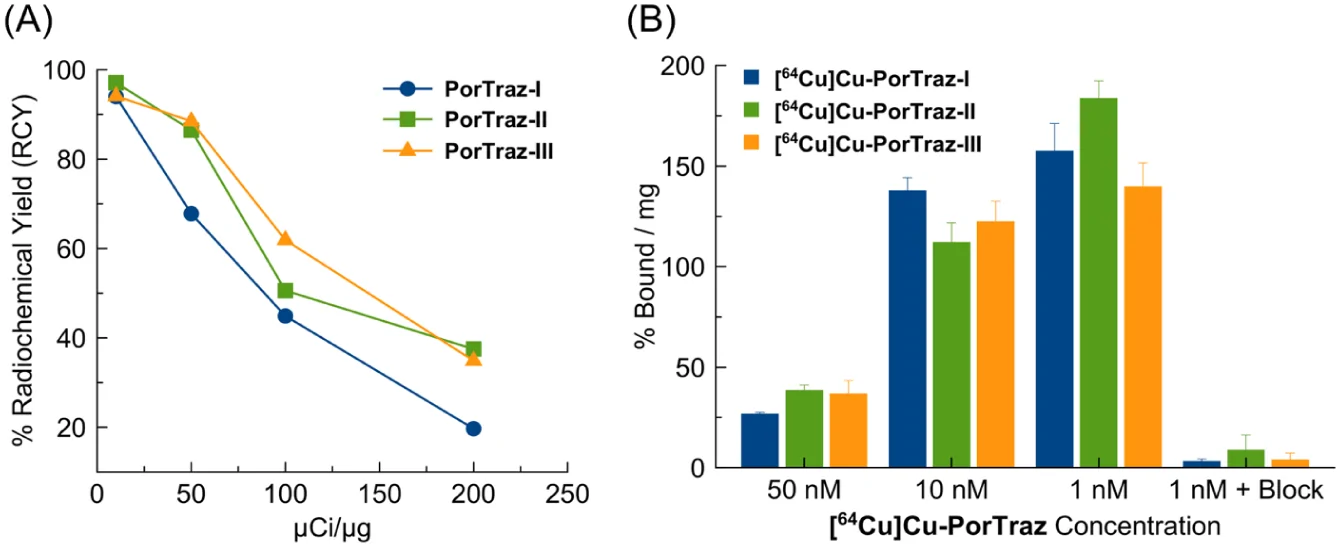
(A) Radiolabeling of **PorTraz** conjugates (*n* = 1). (B) *In vitro* [**^64^Cu]­Cu-PorTraz** binding study with BT-474 HER2+ cancer cells (*n* = 3).

The three radiolabeled **[** ^**64**^ **Cu]­Cu-PorTraz** conjugates were examined for HER2 binding *in vitro*. HER2+ cancer cells (BT-474 cell line) were incubated at several **[** ^**64**^ **Cu]­Cu-PorTraz** concentrations. The results are shown in Figure 4B. The percentage bound per milligram of protein present increased with decreasing concentration of **[** ^**64**^ **Cu]­Cu-PorTraz**. Nearly all binding was lost with the inclusion of a blocking dose of 850 nM of unmodified trastuzumab. An increase in percentage bound per milligram of protein indicates desirable binding of **[** ^**64**^ **Cu]­Cu-PorTraz** conjugates to HER2+ cells, while the loss of binding in the presence of a blocking dose of unmodified trastuzumab indicates specificity for the HER2 receptor. Furthermore, the binding to HER2+ cells of the three **[** ^**64**^ **Cu]­Cu-PorTraz** constructs was not significantly different at the 95% confidence interval. This cell-binding study indicates that (1) **[** ^**64**^ **Cu]­Cu-PorTraz** has a high uptake in HER2+ cancer cells and (2) **[** ^**64**^ **Cu]­Cu-PorTraz** binds to the HER2 membrane receptor with differing numbers of conjugated porphyrins over the range explored (2.3–5.2).

Given the desirable results *in vitro*, the porphyrin–trastuzumab constructs were examined in two *in vivo* studies (Figures 5 and 6). **PorTraz-V** was prepared using a reactant ratio of 8:1 porphyrin-to-trastuzumab and was purified to have an average of 4.1 porphyrins per trastuzumab (Figure S5). **PorTraz-V** was radiolabeled at a specific activity of 20 μCi/μg and showed 98.6% radiochemical purity by instant thin-layer chromatography^45^ (iTLC). Mice bearing HER2+ (BT-474) and HER2– (MDA-MB-468) tumor xenografts were injected with 5 μg of **[** ^**64**^ **Cu]­Cu-PorTraz-V** via the tail vein. At 21 h post-injection, the mice were imaged using PET/CT (Figure 5A and B), then sacrificed for analysis of the biodistribution of the porphyrin–trastuzumab conjugate (Figure S6). Figure 5C shows preferential tumor uptake in the HER2+ tumor, indicating specific tumor targeting based on the SUV (*p* < 0.01). Analysis of the biodistribution of **[** ^**64**^ **Cu]­Cu-PorTraz-V** showed tumor accumulation was significantly increased in the HER2+ group (*p* < 0.0001), while the rest of the biodistribution was similar to that in the HER2– group, indicating specific HER2 targeting by **[** ^**64**^ **Cu]­Cu-PorTraz-V** (Figure S6).

**Figure 5:**
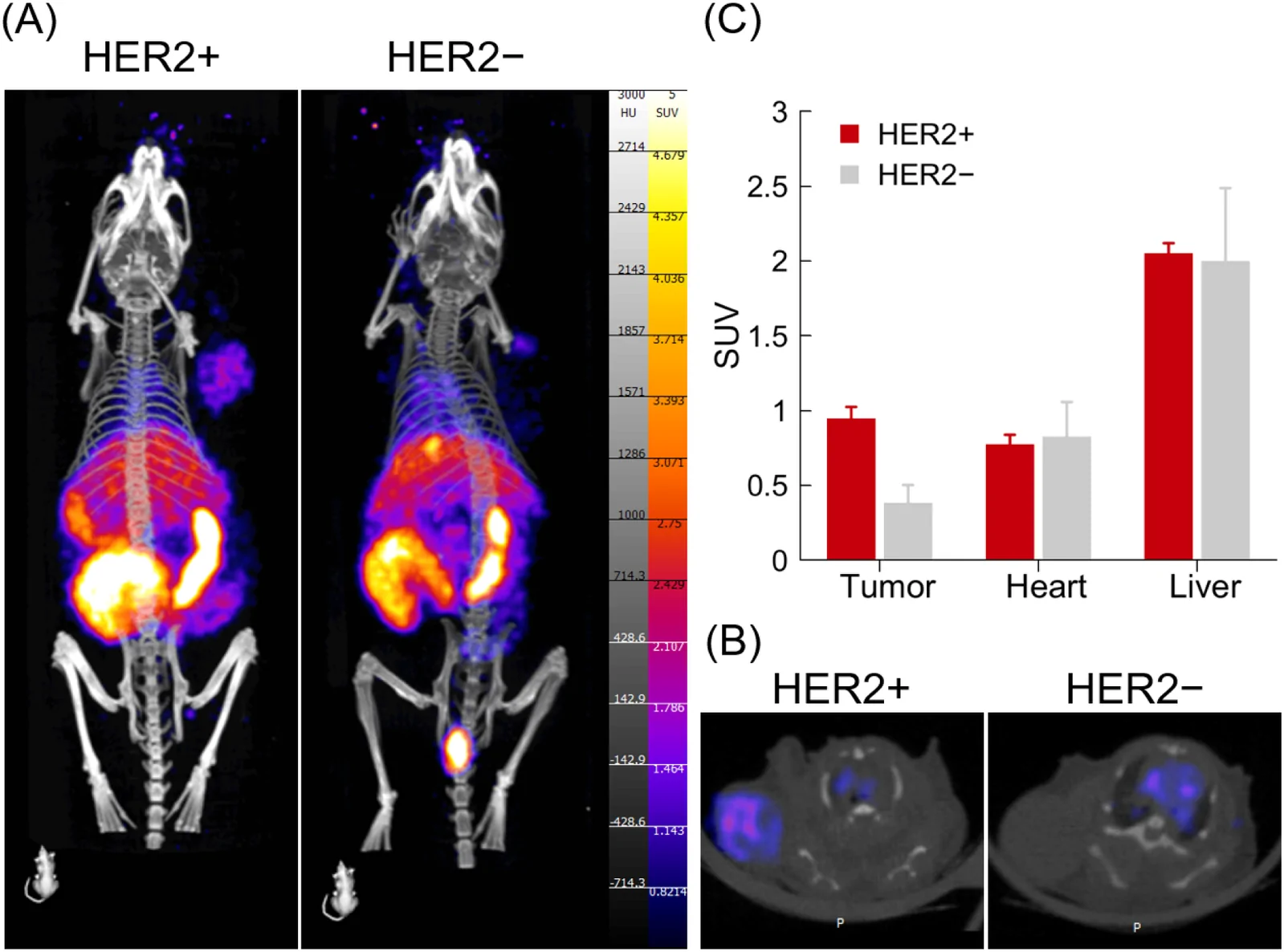
PET/CT imaging of **[^64^Cu]­Cu-PorTraz-V** in HER2+ (*n* = 4) and HER2– (*n* = 3) tumor-bearing mice at 21 h post-injection: (A) coronal view, and (B) transverse view. (C) SUV results for HER2± tumors.

**Figure 6:**
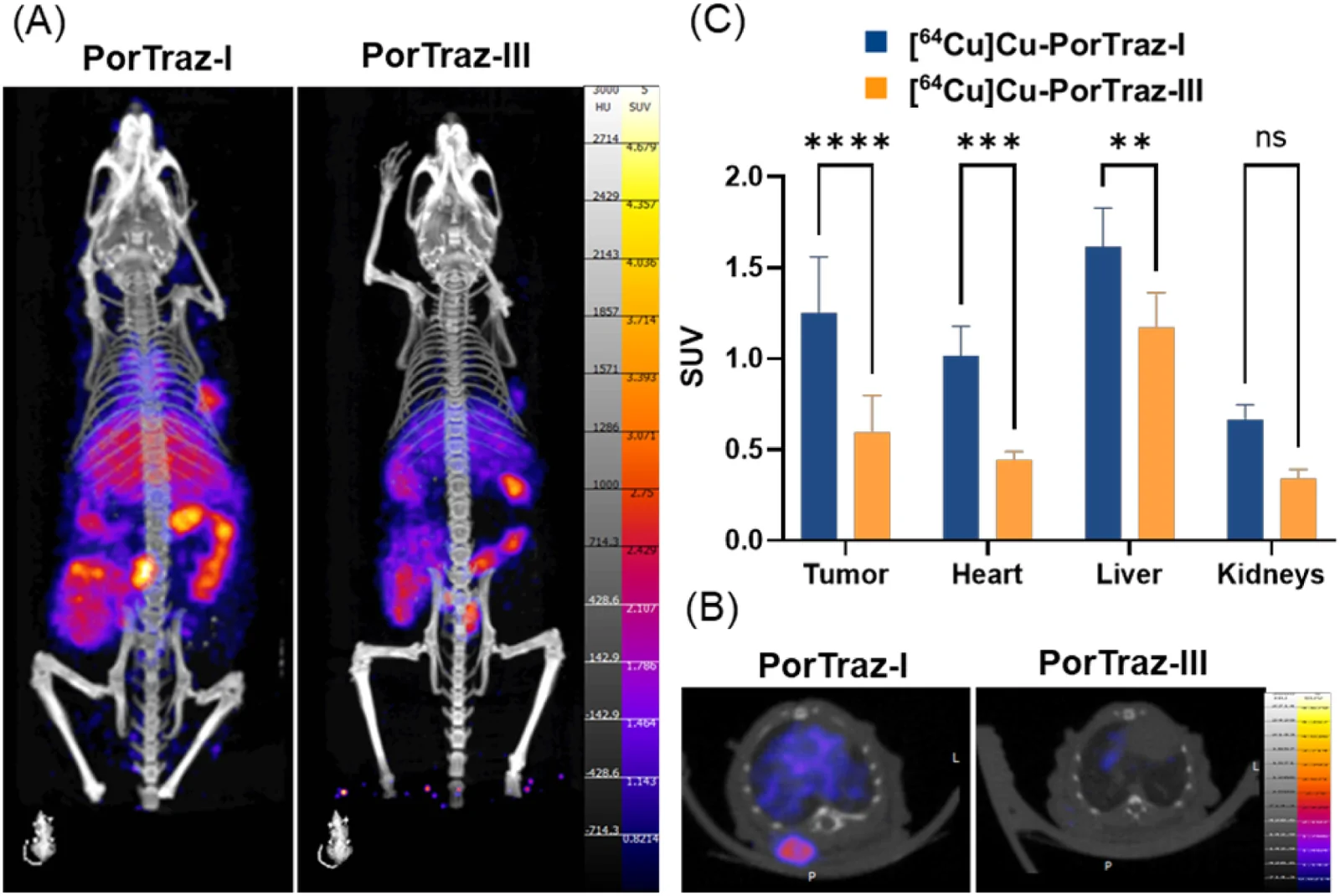
PET/CT imaging of **[^64^Cu]­Cu-PorTraz-I** and **[^64^Cu]­Cu-PorTraz-III** in BT-474 (HER2+) tumor-bearing mice at 24 h (*n* = 4 per group): (A) coronal view, and (B) transverse view. (C) SUV results of **PorTraz** with different conjugation ratios.

Additional *in vivo* experiments examined the properties of the **PorTraz** conjugates bearing different numbers of porphyrin chelators. **PorTraz-I** and **PorTraz-III,** with different average porphyrin-to-trastuzumab conjugation ratios (2.3 and 5.2, respectively), were radiolabeled at a specific activity of 10 μCi/μg. A sample (5 μg) of each **[** ^**64**^ **Cu]­Cu-PorTraz-I** and **[** ^**64**^ **Cu]­Cu-PorTraz-III** was injected via the tail vein. At 24 h post-injection, the mice were imaged using PET/CT (Figure 6A and B), then euthanized for biodistribution analysis (Figure S7). SUV analysis in Figure 6C showed significantly greater tumor accumulation after 24 h of **[** ^**64**^ **Cu]­Cu-PorTraz-I** (1.2 ± 0.3) compared to that of **[** ^**64**^ **Cu]­Cu-PorTraz-III** (0.6 ± 0.2) (*p* < 0.001), whereas **[** ^**64**^ **Cu]­Cu-PorTraz-V** (0.9 ± 0.1) gave results not significantly different from either. Higher SUV results of **[** ^**64**^ **Cu]­Cu-PorTraz-I** were also observed in the heart (*p* < 0.001) and liver (*p* < 0.01) at 24 h compared to those of **[** ^**64**^ **Cu]­Cu-PorTraz-III**. Biodistribution analysis showed mainly nonsignificant reductions of **PorTraz-III** in many organs at 24 h compared to **PorTraz-I**, and small but statistically significant reductions in the blood and large intestine (Figure S7). The modest decreases in tumor and off-target accumulation of **PorTraz-III** may be the result of alterations in clearance rate, but additional studies would be required to confirm this.

As HER2 is such an important target in oncology, several approaches have been developed for imaging this receptor, including radiolabeled intact antibodies as described here, radiolabeled peptides, antibody fragments, nanobodies, and minibodies.^46^ The antibody approach tends to lead to overall higher absolute tumor uptake, albeit at later time points post-imaging, whereas the others generally show adequate tumor-to-background ratios for visualization at earlier time points.^47,48^ An important advantage of using the FDA-approved intact antibody is that the toxicity profile is well-known, providing a clear path to clinical translation.^49–51^ Additionally, the use of copper-64 for imaging results in a lower radiation dose to patients compared to longer-lived radionuclides such as zirconium-89.

As recently reported, porphyrin macrocycles exhibit high specificity for copper-64,^28^ which may prove to be attractive for use in radiotheranostics. The absolute tumor uptake in the current study is somewhat lower than that reported in several studies, but this may be the result of the use of different animal models with varying levels of HER2 expression.^52^ In a recent report by Woo et al.,^53^ a ^64^Cu-NOTA-Trastuzumab conjugate in the BT-474 model gave tumor-to-blood and tumor-to-muscle ratios similar to those reported herein; on the other hand, data from the current study illustrate a higher signal for the large intestine. Further studies of porphyrins with distinct solubilizing groups may shed light on the impact of molecular design on biodistribution.

## Conclusions

A sparsely substituted bioconjugatable porphyrin was combined with trastuzumab to afford porphyrin–trastuzumab products with a range of conjugation ratios (2.3–5.2). The conjugates were analyzed (by UV–vis spectroscopy, MALDI-MS, and CE-MS) and radiolabeled with copper-64 at a molar activity of up to 111 MBq/nmol with >90% radiochemical yield. Conjugates with varying average numbers of porphyrin chelators per antibody displayed similar immunoreactivity *in vitro* toward BT-474 (HER2+) cells. One porphyrin–trastuzumab conjugate examined with BT-474 (HER2+) cells *in vivo* exhibited significant uptake when compared to MDA-MB-468 (HER2−) cells (*p* < 0.0001). Two other porphyrin–trastuzumab conjugates (conjugation ratios 2.3 vs 5.2) showed a decrease in SUV for tumor, heart, and liver for the conjugate bearing more porphyrin chelators per antibody. The results suggest the utility of designer porphyrins for use in radiotheranostics.

## Methods

### General Methods

All characterization methods were performed at room temperature. Silica (SiliaFlash P60, 40–63 μm, Silicycle, R12030B) and C_18_-reversed phase silica (C18 60A, 40–63 μm, Silicycle, R33230B) were used for column chromatography. Commercial compounds were used as received unless noted otherwise. Noncommercial, known compounds dipyrromethane **1**,^30^ **2**,^31^ and **3** ^27^ were prepared as described in the literature. The synthesis procedures and characterization data for the two porphyrins and the various porphyrin–trastuzumab conjugates are provided in the Supporting Information.

### UV–Vis Absorption Spectroscopy

Samples were analyzed by UV–vis absorption spectroscopy using three instruments: a Nanodrop (Nanodrop One Spectrophotometer), a standard benchtop UV–vis instrument (Agilent Cary 8453 Spectrophotometer), and the DAD to monitor the eluent of an HPLC (HP Agilent 1100). On the Nanodrop, 2 μL of each sample was analyzed using the automated path length setting, which alters the path length of the instrument to avoid saturation of the detector. On the benchtop spectrophotometer, samples were analyzed in a 1 cm quartz cuvette at concentrations ranging from 1–10 μM. On the HPLC-DAD, the UV–vis spectrum of the eluent was integrated over the entire peak (from 8–12 min; Figures S3 and S5). At this retention time, the eluent system is comprised of ∼50:50 acetonitrile/H_2_O. Regardless of the composition of acetonitrile/H_2_O, the porphyrin molar absorption coefficient was essentially unchanged (Figure S1). The concentration of porphyrin was determined using the molar absorption coefficient (250,000 M^–1^cm^–1^) at the Soret maximum, which appeared in the region 400–410 nm depending on the medium and the instrument.

### Multicomponent Analysis

The concentration of the porphyrin can be determined directly from the absorbance of the Soret band (ε = 250,000 M^–1^cm^–1^) as the porphyrin alone absorbs in this region. Then, using the known concentration of porphyrin, analysis is performed on the peak at 280 nm to calculate the concentration of the trastuzumab antibody (ε = 225,000 M^–1^cm^–1^)^40^ after subtracting for the absorption of the porphyrin (ε = 6,000–8,000 M^–1^cm^–1^ depending on the medium; see the Supporting Information). The concentrations of porphyrin and antibody in the purified conjugates were used to calculate the conjugation ratio.

### MALDI-MS

The analysis of porphyrin–antibody conjugates was carried out by MALDI-MS using a Bruker Autoflex maX instrument. A saturated solution of sinapinic acid in 50/50 acetonitrile/H_2_O w/0.1% formic acid was used as the matrix. A 1.0 μL drop of the **PorTraz** sample in a volatile ammonium buffer (50 mM NH_4_HCO_3_ or NH_4_OAc) was spotted onto the plate, then a 1.5 μL drop of matrix was added. The 2.5 μL of liquid was mixed with a mechanical pipette, then allowed to dry before analysis. The mass range was set between 100 kDa and 160 kDa.

### HPLC

The purity of each **PorTraz** sample was examined using a Hewlett-Packard Series 1100 HPLC fitted with a ThermoFisher (Waltham, MA) MAbPac Phenyl RP column, employing a gradient of 20–80% acetonitrile (containing 0.1% TFA) in water (containing 0.1% TFA) over 20 min at a flow rate of 0.5 mL/min. The chromatography column was contained in an oven set at 80 °C. The DAD, located outside the oven, was used to monitor the absorbance at 280 and 405 nm. The trastuzumab antibody and each **PorTraz** sample eluted after ∼8 min, based on the chromatogram with detection at 280 nm.

### CE-MS

Samples were analyzed at the Metabolomics and Proteomics Core at the University of North Carolina – Chapel Hill. Samples were provided at a concentration of 0.5–1 mg/mL in ammonium acetate (50 mM, pH 4.75). The samples were diluted 1:1 in peptide background electrolyte (BGE), which was comprised of 49.5% acetonitrile, 49.5% water, and 1% formic acid; pH ∼ 2.4. The samples were analyzed on a Thermo QExactive HF equipped with a 908 Devices ZipChip for CE separation. Byos software was used to deconvolute each mass spectrum. The mass spectra data were digitized and replotted from an original report from the UNC facility, as described previously.^54^

### Preparation, Purification, and Characterization of Porphyrin–Trastuzumab Conjugates^26^

The conjugation reaction was carried out by the addition of 1 part of a stock solution of **Por-NHS** (∼50 mM) in DMSO to 19 parts of a stock solution of trastuzumab (130 μM) in aq carbonate. This resulting reaction mixture, containing 5% DMSO and 47.5 mM aq carbonate, was placed on a rotating platform in an incubator at 37 °C for 2 h. The crude mixture was passed through a spin desalting column, which removed porphyrin species (unreacted **Por-NHS**, hydrolyzed product **Por-CO**_**2**_ **H**, as well as other porphyrin byproducts) and also facilitated a buffer exchange to aq HEPES, the desired buffering system for radiolabeling. The preparation of each porphyrin–trastuzumab sample (**PorTraz-I**, **II**, **III**, **IV**, and **V**) is described in the Supporting Information.

### Radiolabeling of a Porphyrin–Trastuzumab with Copper-64 ^26^

Copper-64 (*t*_1/2_ = 12.7 h) was purchased from the UAB Cyclotron Facility. Copper-64 in 0.1 M HCl was neutralized and combined with a **PorTraz** sample at a ratio of 10–200 μCi/μg in aq HEPES. The reaction mixture was incubated at 37 °C for 30 min with shaking at 500 rpm. Radiochemical purity was evaluated by running 1 μL of the reaction mixture on iTLC paper in 50 mM diethylenetriaminepentaacetate. Under these conditions, free copper-64 runs with the solvent, and the **[** ^**64**^ **Cu]­Cu-PorTraz** product remains at the baseline.

### Cell Culture/*In Vitro* ^26^

BT-474 cells were grown in DMEM supplemented with 10% FBS and 1.8 mM insulin at 37 °C in an atmosphere of 5% CO_2_. For cell uptake studies, 250,000 BT-474 cells were seeded into each well in a 12-well plate 24 h before the study. On the day of the study, the media was removed via aspiration and replaced with 400 uL of complete media containing an indicated amount of **[** ^**64**^ **Cu]­Cu-PorTraz** or blocked with 850 nM of unmodified trastuzumab. Cells were incubated at 37 °C for 1 h before the media was removed. Cells were washed twice with 1 mL of cold phosphate-buffered saline (PBS). Cells were removed from the plate using 200 μL of 0.2 M NaOH and collected for gamma counting (Hidex, Turku, Finland). Uptake was normalized to protein content using a BCA assay (Pierce, Thermo Fisher, MA).

### Mouse Model/*In Vivo* ^26^

Female athymic nude mice were purchased from Jackson Laboratories. All animal procedures were approved by the University of Alabama at Birmingham IACUC under animal protocol number IACUC-22773. Mice were subcutaneously implanted with 700,000 BT-474 or MDA-MB-468 cells in Matrigel:PBS (1:1), and mice with BT-474 tumors also received house-made^47^ estradiol pellets. The mice were used for imaging studies 3–4 weeks after tumor implantation.

For PET imaging, mice were injected with 52.9 ± 8.9 μCi/mouse of a **[** ^**64**^ **Cu]­Cu-PorTraz** sample. Mice were imaged at 21–24 h postinjection on a Sofie GNEXT PET/CT using a 25-min PET acquisition and an 80 kVp CT. After imaging, mice were euthanized, organs were collected, and gamma counting was performed on a Hidex gamma counter. The data were decay-corrected and used to calculate the percent injected dose per gram of tissue (%ID/g). Image processing was performed using VivoQuant software (Invicro, now Perceptive) to obtain SUV results for select tissues.

## Supplementary Material



## Notes

Synthesis experimental procedures; preparation of porphyrin–trastuzumab conjugates; absorption spectra for porphyrins in various media and determination of the molar absorption coefficient for **Por-CO**_**2**_ **H**; HPLC traces of porphyrin–trastuzumab conjugates; tissue biodistribution data; and ^1^H and ^13^C­{^1^H} NMR spectra for all new compounds (PDF)
