# Supplementary material for: Designer 64Cu Porphyrin–Trastuzumab Conjugates for Imaging of HER2-Positive Breast Cancer
Source: Chem Biomed Imaging. 2026 Jun 9;4(10):2216–25. doi: 10.1021/cbmi.6c00024 (PMC13715361; doi:10.1021/cbmi.6c00024)
Supplement: Supplementary file 1 [file im6c00024_si_001.pdf]

Supporting Information for:

Designer ^{64}Cu Porphyrin–Trastuzumab Conjugates for Imaging of HER2-Positive Breast Cancer

Peter C. Lawing,[a] Hailey A. Houson,[b] Zhiyuan Wu,[c] Quang-Thien Ngo,[a]
Jonathan S. Lindsey,[a,*] and Suzanne E. Lapi[b,*]

[a] Department of Chemistry, North Carolina State University, Raleigh, NC 27695-8204, USA

[b] Department of Radiology, Heersink School of Medicine, University of Alabama at Birmingham, Birmingham, AL 35294, USA

[c] Oncurie, Inc., Raleigh, NC 27608

*Authors to whom correspondence should be sent (lapi@uab.edu and jslindse@ncsu.edu)

Table of Contents

Topic	Page
(1) Chemical synthesis	S2
(2) Porphyrin–trastuzumab conjugates	S3
(3) Measurement of the molar absorption coefficient of **Por-CO$_2$H**	S7
(4) Analysis of porphyrin–trastuzumab conjugates	S8
(5) NMR spectral data	S12

(1) Chemical Synthesis

Bis(2-(trimethylsilyl)ethyl) 4-(15-(4-(((2,5-dioxopyrrolidin-1-yl)oxy)carbonyl)phenyl)porphyrin-5-yl)heptanedioate (4). Following a reported method[27] with some modifications, a solution of **1** (26 mg, 0.18 mmol), **2** (22 mg, 89 μmol), and **3** (38 mg, 87 μmol) in CH_2Cl_2 (8.8 mL) was stirred at room temperature. After 5 min, TFA (24 μL, 0.31 mmol) was added, and the reaction mixture was stirred at room temperature for 30 min. Then, DDQ (60 mg, 0.26 mmol) was added. The resulting mixture was stirred at room temperature for 15 min, and then triethylamine was added (50 μL, 0.36 mmol). After stirring for 15 min, the solution was concentrated under reduced pressure. The crude product comprised of three porphyrins was chromatographed on a single column [silica, CH_2Cl_2/ethyl acetate (50:1)] to obtain the desired *trans*-AB-porphyrin as a red solid (12 mg, 16%): ^{1}H NMR (700 MHz, $CDCl_3$) δ 10.32 (d, J = 17.6 Hz, 2H), 9.80 (d, J = 4.6 Hz, 1H), 9.67 (d, J = 5.6 Hz, 1H), 9.49 (d, J = 4.5 Hz, 1H), 9.47 (d, J = 4.6 Hz, 1H), 9.42 (dd, J = 9.3, 4.5 Hz, 2H), 9.00 (dd, J = 4.5, 2.6 Hz, 2H), 8.62–8.59 (m, 2H), 8.44–8.41 (m, 2H), 5.51 (tt, J = 10.8, 5.5 Hz, 1H), 3.93–3.84 (m, 4H), 3.39–3.33 (m, 2H), 3.20–3.15 (m, 2H), 3.03 (d, J = 27.3 Hz, 4H), 2.39–2.33 (m, 2H), 2.17–2.13 (m, 2H), 0.63–0.52 (m, 4H), –0.17 (s, 18H), –2.83 (d, J = 40.5 Hz, 2H); $^{13}C\{^1H\}$ NMR (175 MHz, $CDCl_3$) δ 173.6, 169.5, 162.3, 149.0, 148.4, 147.1, 146.9, 146.6, 145.32, 145.26, 144.4, 143.6, 135.4, 132.9, 132.7, 132.54, 132.50, 130.5, 130.0, 129.4, 129.0, 128.6, 124.5, 120.5, 116.5, 105.8, 105.3, 62.5, 44.4, 36.3, 33.7, 26.0, 17.1, –1.6; λ_{abs} (DMSO) 408, 504, 539, 575, 630 nm; MALDI-MS obsd 886.42, calcd 886.37 [$(M + H)^+$]; ESI-MS obsd 886.3651, calcd 886.3662 [$(M + H)^+$], M = $C_{48}H_{55}N_5O_8Si_2$.

4-(15-(4-(((2,5-Dioxopyrrolidin-1-yl)oxy)carbonyl)phenyl)porphyrin-5-yl)heptanedioic acid (Por-NHS). Following a reported method[35] with modification, a solution of **4** (14 mg, 16 μmol) in CH_2Cl_2 (1.0 mL) was treated with TFA (600 μL) at room temperature. The reaction mixture was stirred for 4 h at room temperature. The solvent was removed under reduced pressure. The crude product was loaded on a C18-silica pad (1 cm x 2 cm dia) and washed with water (40 mL) followed by acetonitrile/THF (8:1, 10 mL). The final eluant upon concentration afforded a red solid (10.5 mg, 97%): ^{1}H NMR (700 MHz, THF-d_8) δ 10.38 (d, J = 15.0 Hz, 2H), 9.92 (d, J = 4.5 Hz, 1H), 9.84 (d, J = 4.6 Hz, 1H), 9.52 (dd, J = 11.5, 4.5 Hz, 2H), 9.46 (dd, J = 11.1, 4.4 Hz, 2H), 9.04 (dd, J = 4.4, 1.5 Hz, 2H), 8.62–8.57 (m, 2H), 8.50–8.45 (m, 2H), 5.59 (tt, J = 10.2, 5.6 Hz, 1H), 3.36–3.29 (m, 2H), 3.20–3.15 (m, 2H), 2.94 (d, J = 23.2 Hz, 4H), 2.45–2.37 (m, 2H), 2.12–2.07 (m, 2H), –2.73 (d, J = 37.8 Hz, 2H); $^{13}C\{^1H\}$ NMR (175 MHz, THF-d_8) δ

174.6, 170.2, 163.1, 150.2, 149.1, 147.9, 147.6, 146.4, 146.2, 145.4, 144.5, 136.3, 133.4, 133.3, 133.2, 133.1, 130.9, 130.5, 129.9, 129.8, 129.7, 125.9, 122.4, 117.1, 106.3, 105.8, 45.3, 37.3, 33.7, 26.5; λ_{abs} (DMSO) 408, 504, 539, 575, 629 nm; MALDI-MS obsd 686.73, calcd 686.22 [(M + H)$^+$]; ESI-MS obsd 684.2104, calcd 684.2100 [(M – H)$^-$], M = $C_{38}H_{31}N_5O_8$. Note: THF was used in the elution to facilitate dissolution of the title compound.

The hydrolyzed byproduct of **Por-NHS** is the porphyrin–triacid 4-(15-(4-carboxyphenyl)porphyrin-5-yl)heptanedioic acid (**Por-CO_2H**),[27] which is shown in Scheme 1.

(2) Porphyrin–trastuzumab conjugates

PorTraz-I

Stock solution. A stock solution of **Por-NHS** was prepared in DMSO at a concentration of 50 mM based on absorption at 408 nm using a molar absorption coefficient of ε = 240,000 $M^{-1}cm^{-1}$. A 1 µL aliquot of the **Por-NHS** stock solution was diluted to 6 µL in DMSO, giving a solution which was 8.3 mM for **Por-NHS** in DMSO.

Stock solution. A stock solution of trastuzumab was prepared in sodium bicarbonate (50 mM, pH 9.0) at a concentration of 130 µM based on absorption at 280 nm using a molar absorption coefficient of ε = 225,000 $M^{-1}cm^{-1}$.

Reaction process. A 5 µL aliquot of the 8.3 mM **Por-NHS** stock solution (28 µg, 42 nmol) in DMSO was added to 95 µL of the stock solution of trastuzumab (1850 µg, 12 nmol) in sodium bicarbonate buffer (50 mM, pH 9). The solution was homogenized with a mechanical pipette, then placed on a rotating platform in an incubator at 37 °C for 2 h to give the crude reaction mixture.

Purification. A spin desalting column (Zeba™ Spin, 40 kDa MWCO, ThermoFisher) was equilibrated with aq HEPES. The crude reaction mixture was loaded onto this size exclusion desalting column (40 kDa MWCO). A 15 µL aliquot of aq HEPES was loaded onto the column per the manufacturer's recommendations for improved protein recovery. The spin desalting column was centrifuged for 2 min at 700g, and the eluate was collected in a 2 mL polypropylene tube. This step served a dual purpose: exchanging the buffer to aq HEPES and separating the conjugate from other porphyrin species in the reaction mixture. A thick violet band was observed on top of the desalting column (residual porphyrin species) while the eluate appeared as a dark red solution. The eluate was loaded onto a second size exclusion desalting column and subjected to the same protocol described above to ensure complete removal of residual porphyrin species. This sample is referred to as **PorTraz-I**.

HPLC-DAD analysis. A 5 µL aliquot of **PorTraz-I** (2 µg/µL, 10 µg) in aq HEPES was injected into a Hewlett Packard Series 1100 HPLC fitted with a ThermoFisher (Waltham, MA) MAbPac™ Phenyl RP column with a gradient of 20–80% acetonitrile (containing 0.1% TFA) in water (containing 0.1% TFA) over 20 min at a flow rate of 0.5 mL/min. The chromatography column was contained in an oven set at 80 °C. The purity of the product was confirmed using a DAD (Hewlett Packard Series 1100), which was located outside the oven. The signals at 280 nm and 405 nm were used to detect the presence of antibody and porphyrin species, respectively, and to calculate the porphyrin-to-antibody ratio.

MALDI-MS analysis. A 1 µL aliquot of **PorTraz-I** (~12 mg/mL) in aq HEPES was diluted 1:8 with ammonium acetate buffer (50 mM, pH 4.8). This solution of **PorTraz-I** in ammonium acetate was used for MALDI-MS analysis using a saturated solution of sinapinic acid in 1:1 acetonitrile (ACN)/H_2O w/ 0.1% TFA as the matrix. A 1.0 µL aliquot of **PorTraz-I** in ammonium acetate (50 mM, pH 8.5) was spotted onto the plate, then a 1.5 µL aliquot of matrix solution was spotted directly on top of the sample, and the two solutions were mixed with a mechanical pipette. MALDI-MS analysis was performed on a Bruker (Billerica, MA) autoflex maX with an *m/z* range set from 100-160 kDa in linear positive ion mode using 1000 shots.

PorTraz-II

Stock solutions. A stock solution of **Por-NHS** was prepared in DMSO at a concentration of 50 mM. A 2 µL aliquot of the **Por-NHS** stock solution was diluted to 6 µL in DMSO, giving a solution which was 17 mM for **Por-NHS** in DMSO. A stock solution of trastuzumab was prepared as described above.

Reaction process. A 5 µL aliquot of the 17 mM **Por-NHS** stock solution (56 µg, 84 nmol) in DMSO was added to 95 µL of the stock solution of trastuzumab (1850 µg, 12 nmol) in sodium bicarbonate buffer (50 mM, pH 9). The reaction proceeded as described above.

Purification. Purification proceeded as described above. This sample is referred to as **PorTraz-II**.

HPLC-DAD analysis. A 5 µL aliquot of **PorTraz-II** (2 µg/µL, 10 µg) in aq HEPES was analyzed in the same manner described above.

MALDI-MS analysis. **PorTraz-II** was diluted 1:8 in ammonium acetate (50 mM, pH 4.8) and this sample was analyzed by MALDI-MS as described above.

PorTraz-III

Stock solutions. A stock solution of **Por-NHS** was prepared in DMSO at a concentration of 50 mM. A 4 µL aliquot of the **Por-NHS** stock solution was diluted to 6 µL in DMSO, giving a solution which was 33 mM for **Por-NHS** in DMSO. A stock solution of trastuzumab was prepared as described above.

Reaction process. A 5 µL aliquot of the 33 mM **Por-NHS** stock solution (112 µg, 168 nmol) in DMSO was added to 95 µL of the stock solution of trastuzumab (1850 µg, 12 nmol) in sodium bicarbonate buffer (50 mM, pH 9). The reaction proceeded as described above.

Purification. Purification proceeded as described above. This sample is referred to as **PorTraz-III**.

HPLC-Absorption spectral analysis. A 5 µL aliquot of **PorTraz-III** (2 µg/µL, 10 µg) in aq HEPES was analyzed in the same manner described above.

MALDI-MS analysis. **PorTraz-III** was diluted 1:8 in ammonium acetate (50 mM, pH 4.8) and this sample was analyzed by MALDI-MS as described above.

Cu-PorTraz-IV (D)

Stock solutions. A stock solution of **Por-NHS** was prepared in DMSO at a concentration of 50 mM. A 3.64 µL aliquot of the **Por-NHS** stock solution was diluted to 10 µL in DMSO, giving a solution which was 18 mM for **Por-NHS** in DMSO. A stock solution of trastuzumab was prepared as described above.

Reaction process. A 10 µL aliquot of the 18 mM **Por-NHS** stock solution (122 µg, 180 nmol) in DMSO was added to 190 µL of the stock solution of trastuzumab (3700 µg, 24 nmol) in sodium bicarbonate buffer (50 mM, pH 9). The reaction proceeded as described above.

Purification. The reaction mixture was split into two aliquots and buffer exchanged to aq HEPES as described above. This sample is hereafter referred to as **PorTraz-IV**.

Metalation. A 25 µL aliquot of the purified **PorTraz-IV** solution (8 mg/mL) was treated with 25 µL of copper (II) chloride in aq HEPES at a concentration of 1 mM. This gave a mixture which contained 10 equivalents of copper (II) per porphyrin moiety. The mixture was left at 37 °C overnight. This sample, hereafter referred to as **Cu-PorTraz-IV** was carried forward without further purification.

Deglycosylation. **Cu-PorTraz-IV** was deglycosylated using a PNGase F Glycan Cleavage Kit (ThermoFisher). A 25 µL aliquot of the **Cu-PorTraz-IV** solution (4 mg/mL) was diluted to

142 µL with HPLC grade water (Sigma Aldrich). Separately, 3 µL of PNGase F solution (25,000 units) was added to a 16 µL aliquot of deglycosylation buffer. The 19 µL enzyme solution was added to the 142 µL solution of **Cu-PorTraz-IV** and left for 4h at 50°C. The reaction mixture was then diluted to 500 µL in ammonium acetate (50 mM, pH 4.8) and purified using a 100 kDa Amicon centrifuge filter. The 500 µL sample was centrifuged at 14,000 G for 5 minutes. The solution retained above the filter was collected, then diluted again to 500 µL in ammonium acetate. This 500 µL sample was centrifuged again at 14,000 G for 10 minutes. The solution retained above the filter (hereafter referred to as **Cu-PorTraz-IV (D)**) was collected and used for CE-MS analysis. The Amicon centrifugal filtration steps served a dual purpose: exchanging the sample to a volatile ammonium acetate buffer for mass analysis and also to remove the PNGase F enzyme.

HPLC-DAD analysis. 100 µg of **PorTraz-IV**, **Cu-PorTraz-IV**, or **Cu-PorTraz-IV (D)** in aq HEPES was analyzed in the same manner described above.

PorTraz-V

Stock solutions. A stock solution of **Por-NHS** was prepared in DMSO at a concentration of 40 mM based on absorption at 408 nm using a molar absorption coefficient of $\varepsilon = 240{,}000\ M^{-1}cm^{-1}$. A 5 µL aliquot of the **Por-NHS** stock solution was diluted to 10 µL in DMSO, giving a solution which was 20 mM for **Por-NHS** in DMSO. A stock solution of trastuzumab was prepared in sodium bicarbonate (50 mM, pH 9.0) at a concentration of 130 µM based on absorption at 280 nm using a molar absorption coefficient of $\varepsilon = 225{,}000\ M^{-1}cm^{-1}$.

Reaction process. A 5 µL aliquot of the 20 mM **Por-NHS** stock solution (66 µg, 96 nmol) in DMSO was added to 95 µL of the stock solution of trastuzumab (1850 µg, 12 nmol) in sodium bicarbonate buffer (50 mM, pH 9). The reaction proceeded as described above.

Purification. Purification proceeded as described above. This sample is referred to as **PorTraz-V**.

HPLC-DAD analysis. A 5 µL aliquot of **PorTraz-V** (2 µg/µL, 10 µg) in aq HEPES was analyzed in the same manner described above.

(3) Measurement of the molar absorption coefficient

Analysis of the porphyrin monomer **Por-CO_2H** at a concentration of 3.36 μM was carried out on a Shimadzu 2600i Spectrophotometer using a 1 cm quartz cuvette. These experiments were performed on samples with a known concentration of porphyrin which allowed for the analysis of the porphyrin molar absorption coefficient in a variety of solvent conditions (Figure S1).

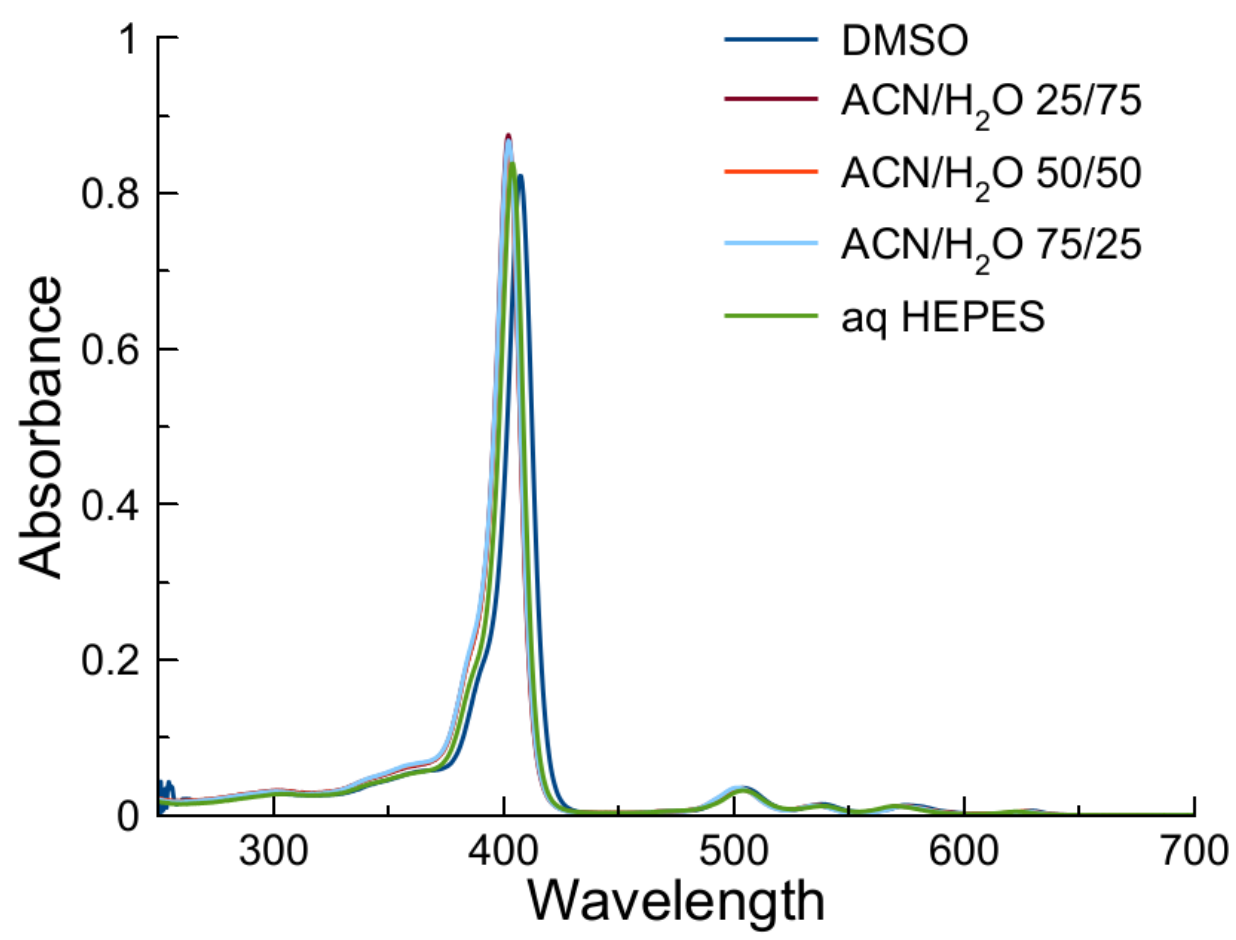

Figure S1. UV-Vis spectra of **Por-CO_2H** in various solvents at room temperature.

The molar absorption coefficient at the λ_{max} (Table S1) were found to be around 250,000 $M^{-1}cm^{-1}$ regardless of solvent conditions, however the λ_{max} did move between 401 and 408 nm in the three solvents analyzed. The absorbance was nearly identical in various mixtures of acetonitrile and water (from 25/75 to 75/25 acetonitrile (ACN)/H_2O). This result indicates that the porphyrin concentration can be determined by the measurement of the Soret band in any of these media.

Table S1. Molar Absorptivity Data for Swallowtail Porphyrin.

Solvent	Molar Absorption Coefficient ($M^{-1}cm^{-1}$) at the given wavelength		
	280 nm	λ_{max} (listed)	505 nm
DMSO	8,000 ± 1,600	250,000 ± 4,000 (407 nm)	11,000 ± 600
ACN/H_2O (50/50)	7,000 ± 1,600	240,000 ± 16,000 (402 nm)	9,000 ± 500
aq HEPES	6,000 ± 500	250,000 ± 1,200 (404 nm)	10,000 ± 200

(4) Analysis of porphyrin–trastuzumab conjugates

Three porphyrin–trastuzumab conjugates (**PorTraz-I**, **II**, and **III**) were analyzed using UV-Vis absorption spectroscopy on three different instruments depending on sample concentrations: (1) Samples from 7-12 mg/mL were directly measured in aq HEPES on a Nanodrop One Spectrophotometer (Figure S2A), which allows for the quantification of concentrated samples without significant sample loss. (2) Samples at 250 µg/mL in aq HEPES were measured in 1-cm quartz cuvettes on an Agilent 8453 Spectrophotometer (Figure S2B). (3) Samples (5 µL at 2 mg/mL) were injected into a high-performance liquid chromatography – diode array detector (HPLC-DAD) instrument, and the UV-Vis spectrum of the eluent was analyzed (Figure S2C), which enables analysis of dilute porphyrin–trastuzumab conjugates without sacrificing large amounts of sample (~10 µg). The calculated results are shown in Table S2. The HPLC traces of three conjugates also confirmed the full removal of unreacted **Por-NHS** and the major byproduct **Por-CO$_2$H** (Figure S3).

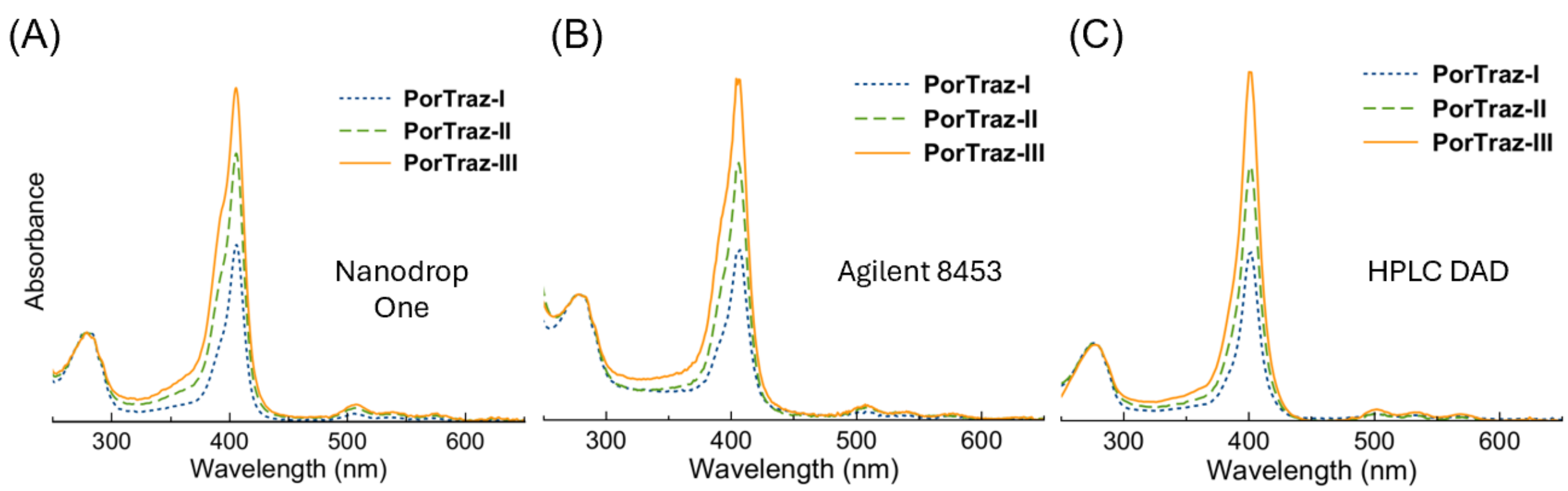

Figure S2. UV Vis-spectra of **PorTraz** conjugates (normalized at the Trastuzumab absorption band at 280 nm with three instruments accommodating a range of sample concentrations (see text): (A) Nanodrop One; (B) Agilent 8453; and (C) HPLC DAD.

Table S2. Conjugation Ratios of Porphyrin per Trastuzumab

**PorTraz** Sample	Ratio in Reaction	Conjugation Ratio		
		Nanodrop	Agilent	DAD
**I**	3.5 : 1	1.8	1.2	2.3
**II**	7.0 : 1	2.8	1.9	3.5
**III**	14 : 1	3.7	2.6	5.2

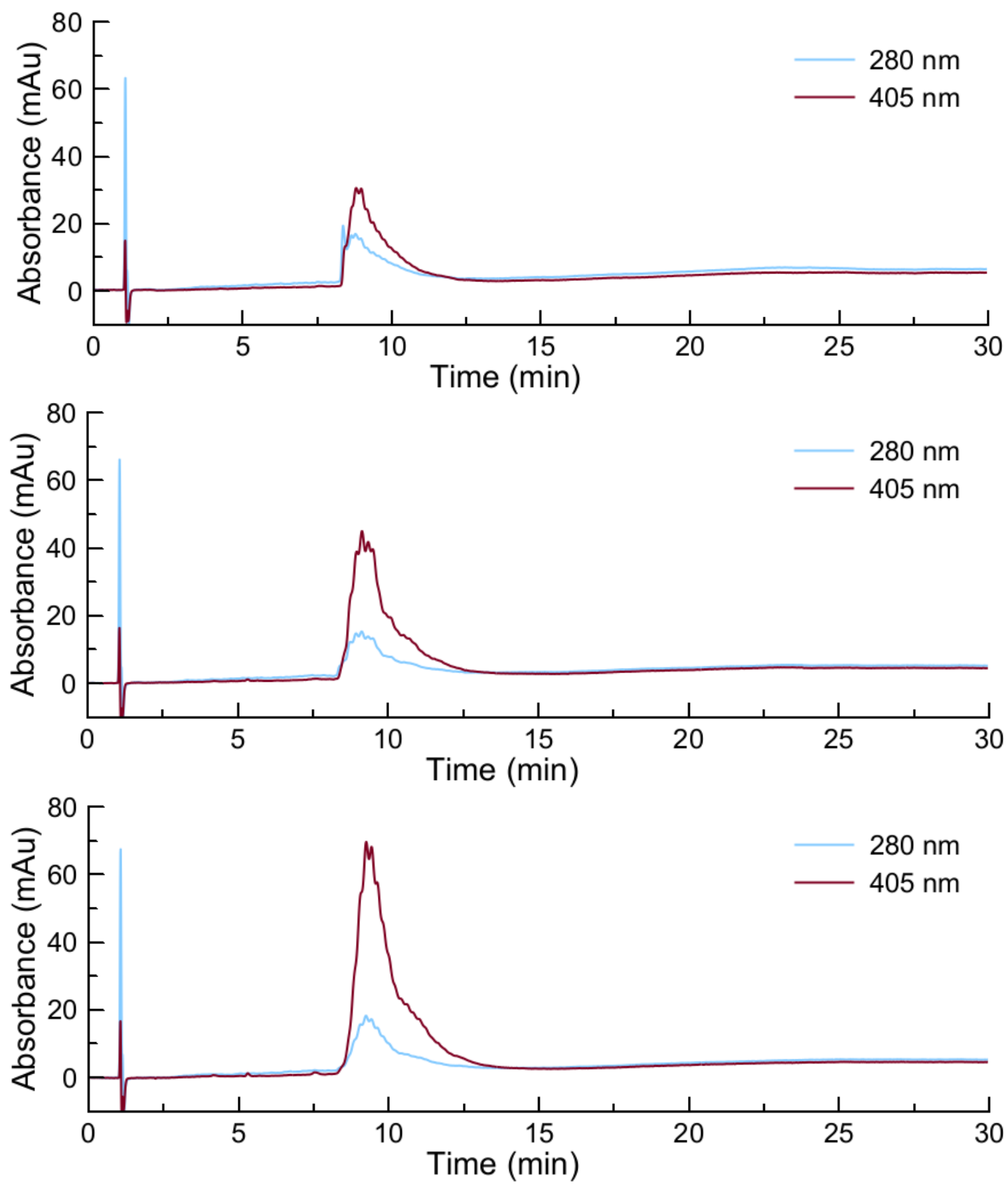

Figure S3. HPLC Chromatogram of **PorTraz-I**, **PorTraz-II**, and **PorTraz-III**.

Some variance in the ratios were observed with the different instruments, which can be explained as follows. First, the baseline in the spectra collected on the Agilent 8453 instrument (Figure S2B) indicates light scattering or absorbance by the HEPES buffer (which is known to tail into the near-UV region). Light-scattering in the UV region is a common problem and must be avoided or corrected,[42] otherwise the signal attributed to trastuzumab at 280 nm is artificially inflated. Second, broadening of the Soret band, given by the increase in the full-width-at-half-maximum (FWHM) observed in both the Agilent 8453 instrument (24 nm) and the Nanodrop instrument (26 nm) compared to the porphyrin alone (13 nm), is suggestive of porphyrin self-aggregation. The decrease in peak height accompanying band broadening lowers the calculated conjugation ratio. On the other hand, the HPLC-DAD shows very little baseline absorbance in the near-UV and maintains a narrow Soret band (FWHM ≈ 17 nm). The improved spectra via the HPLC-DAD likely stems from the decreased concentration of samples (~50 nM compared to >1 μM). The results for each sample are tabulated in Table S3. On the basis of the HPLC-DAD

results, the average conjugation ratio of **PorTraz-I**, **II**, and **III** is hereafter considered to be 2.3, 3.5, and 5.2, respectively.

Table S3. UV-Vis Spectroscopy for Analysis of Porphyrin–Trastuzumab Conjugates

Molecule	Solvent	Instrument	FWHM (nm)	Concentration
**Por-CO_2H**	DMSO	Shimadzu Benchtop	13	3.36 μM
**Por-CO_2H**	ACN/H_2O	Shimadzu Benchtop	13.5	3.36 μM
**Por-CO_2H**	aq HEPES	Shimadzu Benchtop	12.5	3.36 μM
**PorTraz-I**	aq HEPES	Agilent Benchtop	22	1.7 μM
**PorTraz-II**	aq HEPES	Agilent Benchtop	23	1.7 μM
**PorTraz-III**	aq HEPES	Agilent Benchtop	25	1.7 μM
**PorTraz-I**	aq HEPES	Nanodrop	18	80 μM
**PorTraz-II**	aq HEPES	Nanodrop	22	80 μM
**PorTraz-III**	aq HEPES	Nanodrop	26	50 μM
**PorTraz-I**	ACN/H_2O	HPLC DAD	17	~50 nM
**PorTraz-II**	ACN/H_2O	HPLC DAD	17	~50 nM
**PorTraz-III**	ACN/H_2O	HPLC DAD	17	~50 nM

The porphyrin–trastuzumab samples and the unconjugated trastuzumab were further analyzed by MALDI-MS. The inherently limited resolution of MALDI-MS precludes detection of conjugates bearing one, two, three, four, etc., porphyrins per antibody (porphyrin, 570 Da; trastuzumab, ~148,500 Da). However, there is a clear shift in mass-to-charge ratio (*m/z*) for the conjugates synthesized from a higher porphyrin–trastuzumab ratio in the reaction, which coheres with the trend of increased conjugation ratio observed by absorption spectroscopy. Additional, peak broadening observed in MALDI-MS at higher conjugation ratios correlates to the presence of a wider variety of conjugates expected based on the Poissonian nature of the mixture.[25]

Table S4. Data from MALDI-MS analysis.

	**Trastuzumab**	**PorTraz-I**	**PorTraz-II**	**PorTraz-III**
***m/z* (kDa)**	148.6	149.6	150.9	151.7
**FWHM (kDa)**	2.7	3.7	4.1	5.3
**Δ *m/z* (Da)**	–	1000	2300	3100
**Porphyrin Chelators**	–	1.8	4.0	5.4
**Ratio in Reaction**	–	3.5	7	14

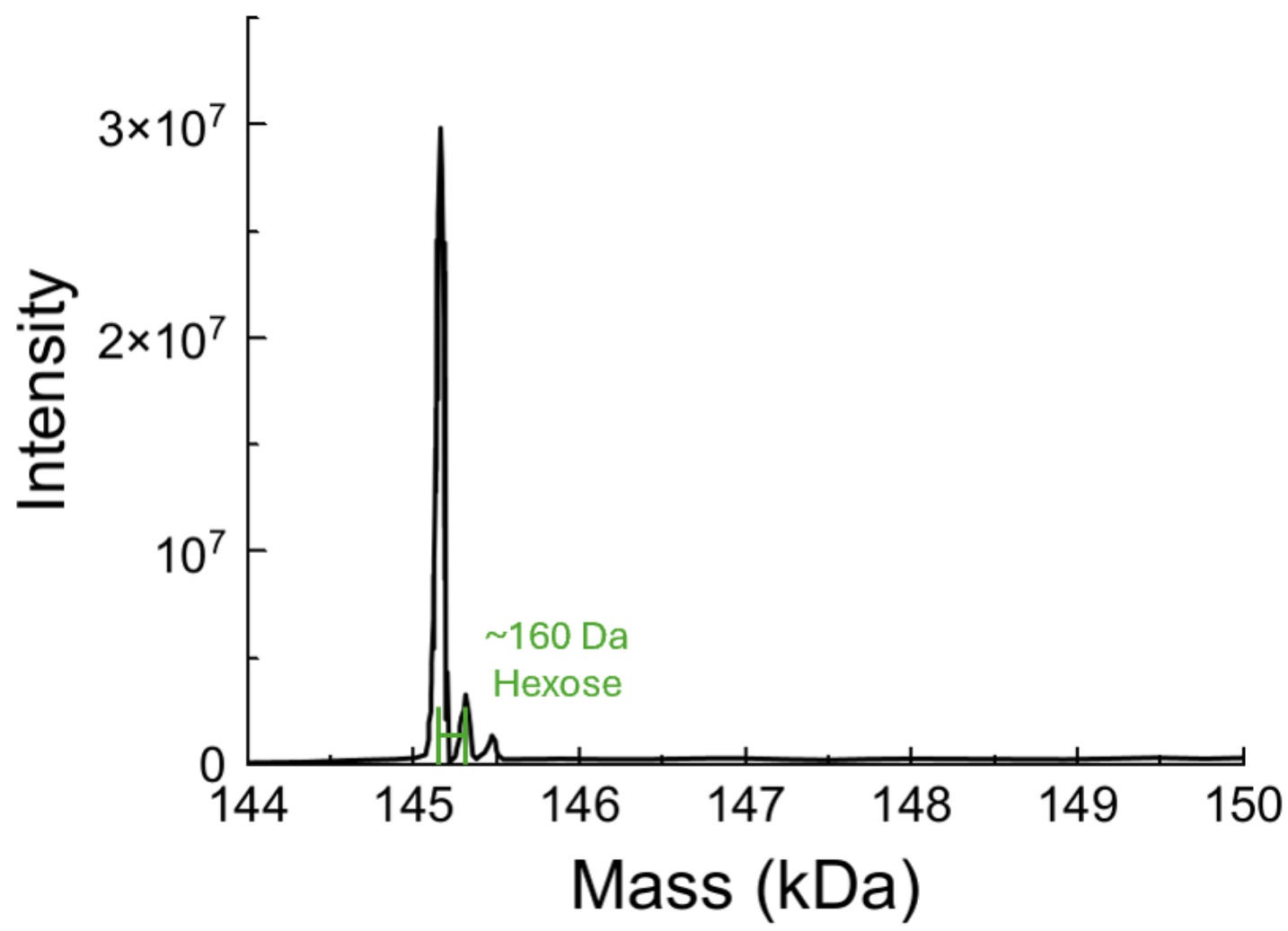

Figure S4. CE-MS results for deglycosylated trastuzumab.

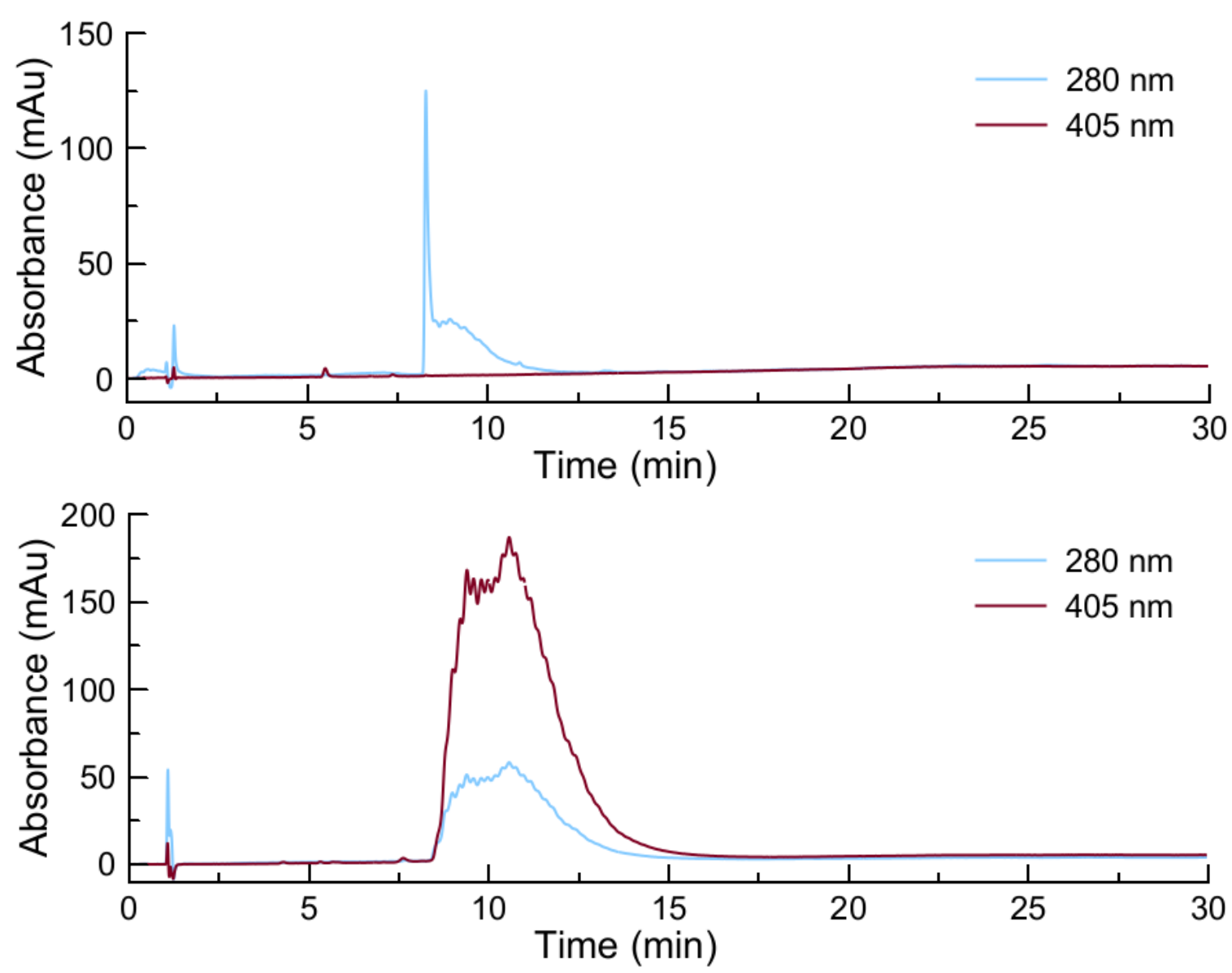

Figure S5. HPLC Chromatogram of trastuzumab and **PorTraz-V**, comfirming the removal of **Por-NHS** and the major byproduct **Por-CO_2H**.

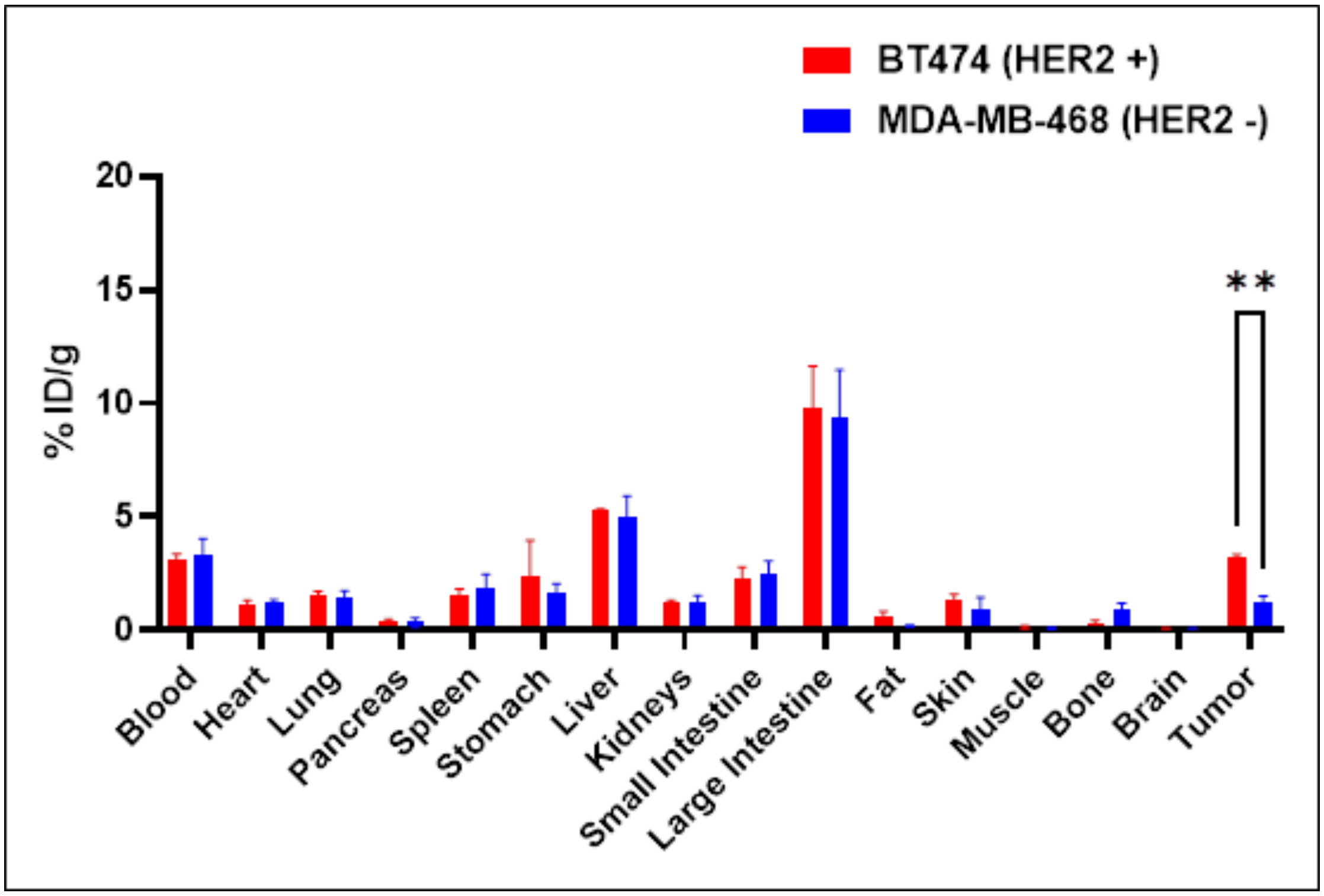

Figure S6. Biodistribution data. Statistical analysis was performed with multiple unpaired t test assuming individual variance and using a two-stage step-up.

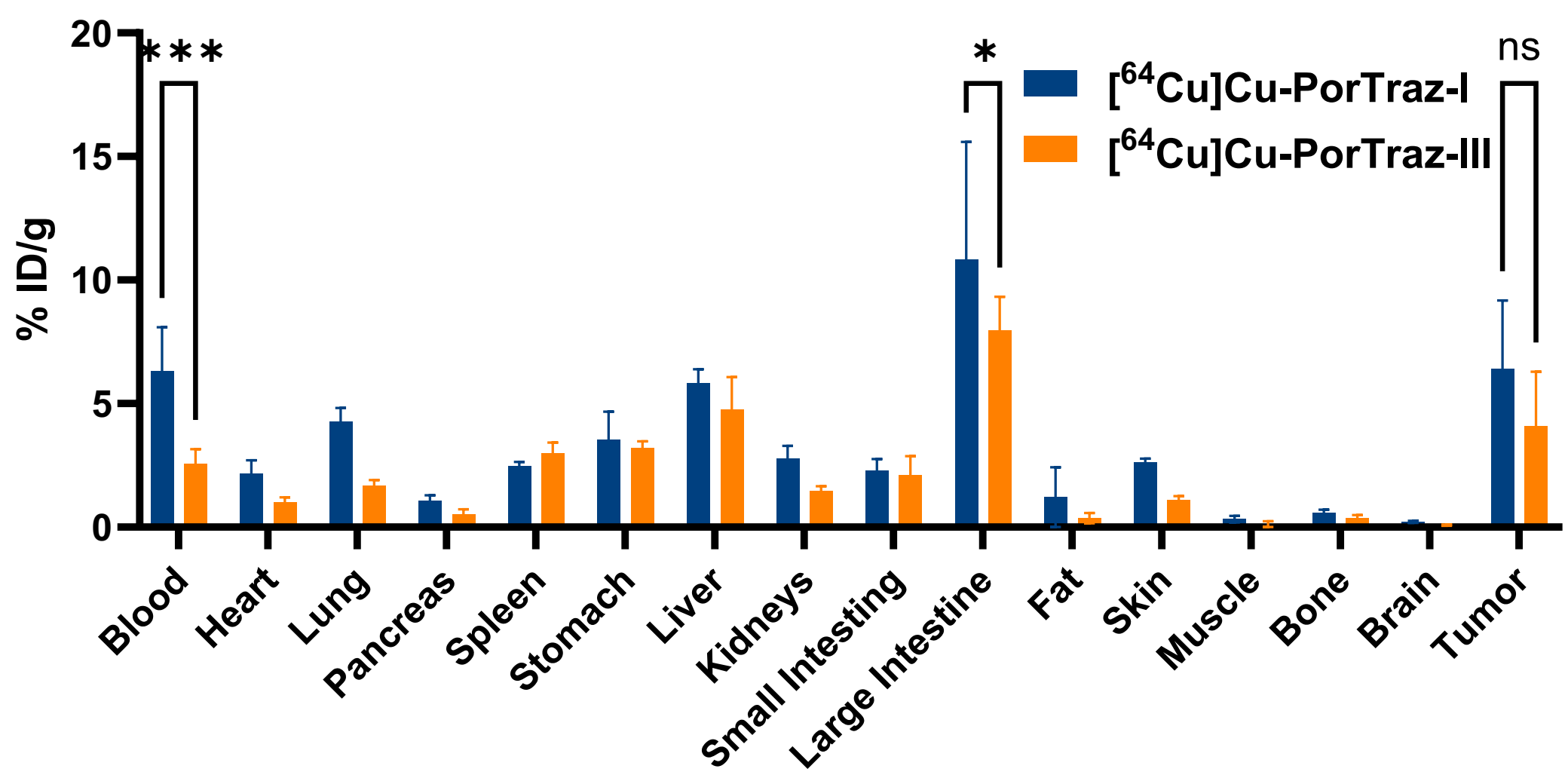

Figure S7. Biodistribution data.

(5) NMR spectral data

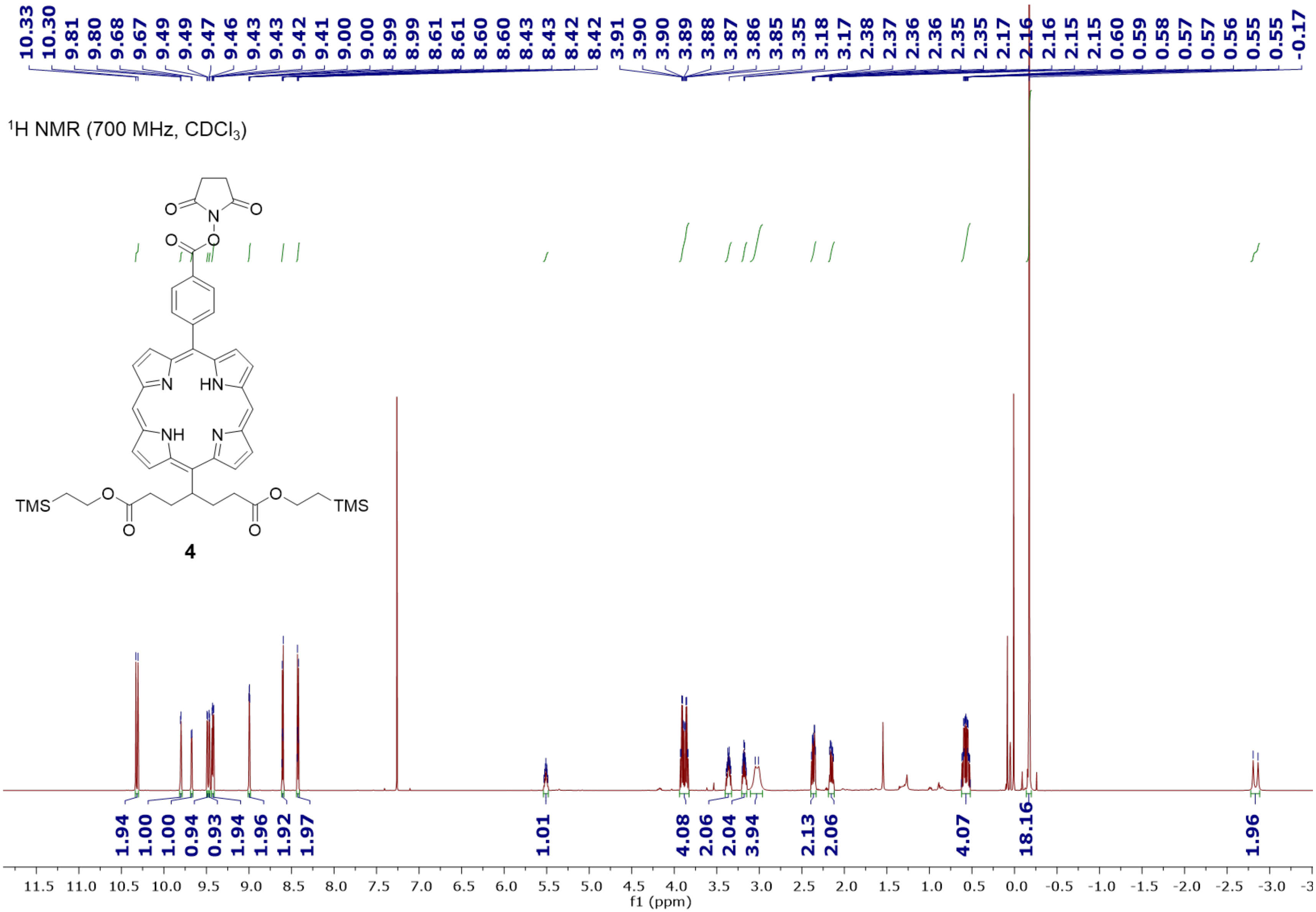

^{1}H NMR spectrum of **4** (in $CDCl_3$)

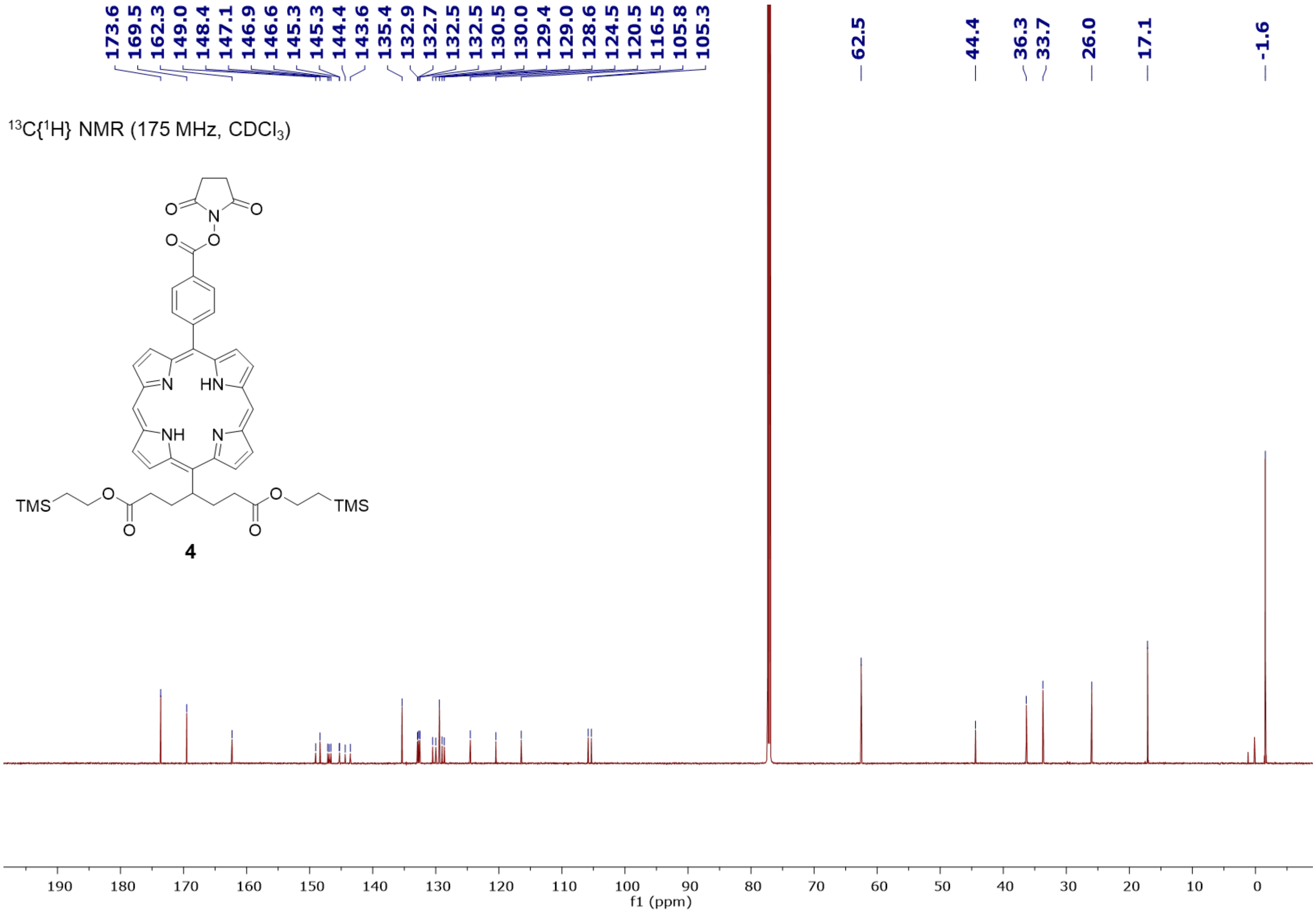

$^{13}C\{^1H\}$ NMR spectrum of **4** (in $CDCl_3$)

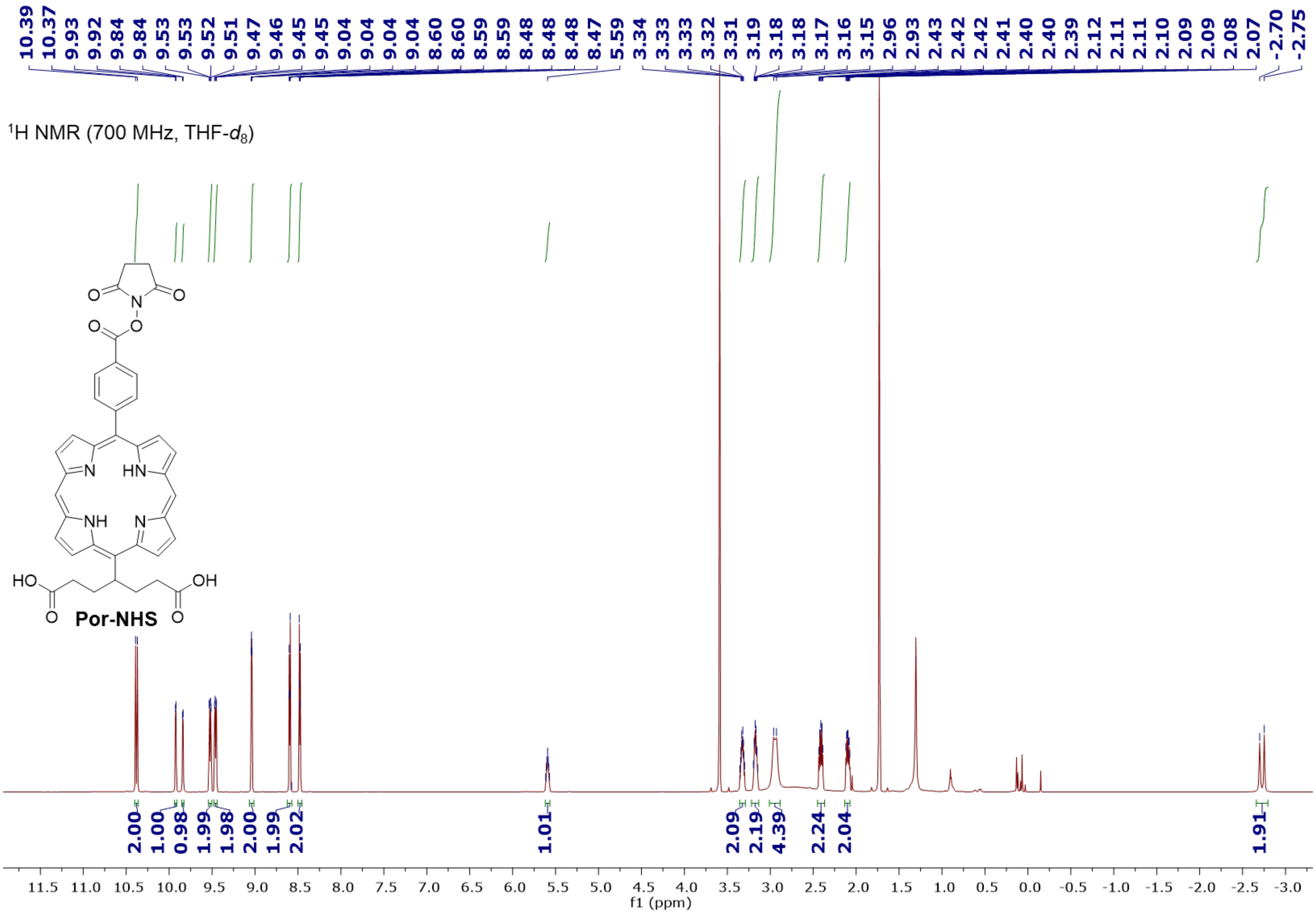

^{1}H NMR spectrum of **Por-NHS** (in THF-d_8)

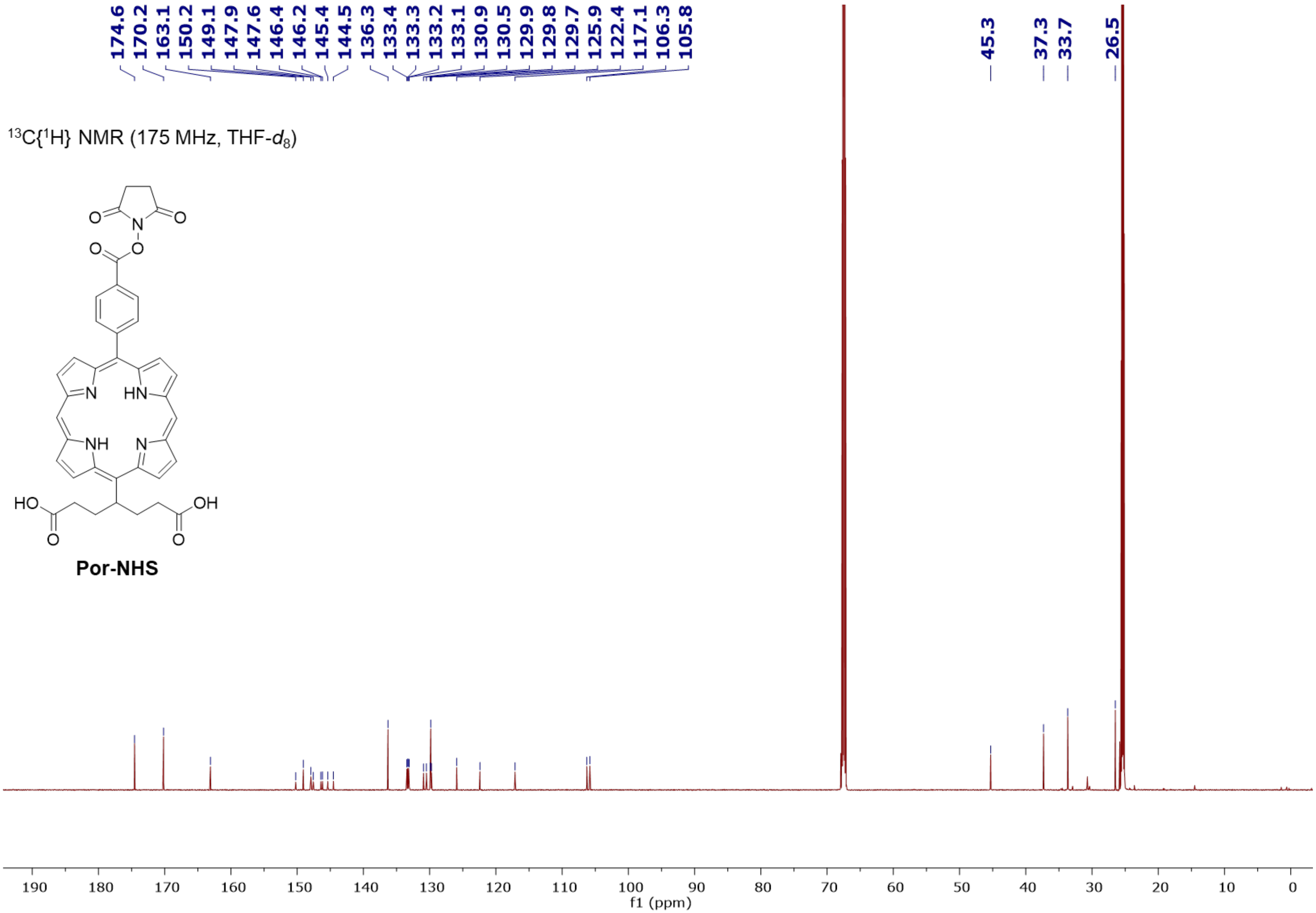

$^{13}C\{^{1}H\}$ NMR spectrum of **Por-NHS** (in THF-d_8)
